# The Transcriptome of SH-SY5Y at Single-Cell Resolution: A CITE-Seq Data Analysis Workflow

**DOI:** 10.3390/mps4020028

**Published:** 2021-05-06

**Authors:** Daniele Mercatelli, Nicola Balboni, Francesca De Giorgio, Emanuela Aleo, Caterina Garone, Federico Manuel Giorgi

**Affiliations:** 1 Department of Pharmacy and Biotechnology, University of Bologna, 40126 Bologna, Italy; nicola.balboni@unibo.it; 2 Department of Medical and Surgical Sciences, University of Bologna, 40138 Bologna, Italy; francesca.degiorgio8@unibo.it (F.D.G.); caterina.garone@unibo.it (C.G.); 3 Center for Applied Biomedical Research (CRBA), University of Bologna, 40138 Bologna, Italy; 4 IGA Technology Services, 33100 Udine, Italy; ealeo@igatechnology.com

**Keywords:** CITE-seq, neuroblastoma, single-cell, transcriptomics, unsupervised learning, gene regulatory networks

## Abstract

Cellular Indexing of Transcriptomes and Epitopes by Sequencing (CITE-seq) is a recently established multimodal single cell analysis technique combining the immunophenotyping capabilities of antibody labeling and cell sorting with the resolution of single-cell RNA sequencing (scRNA-seq). By simply adding a 12-bp nucleotide barcode to antibodies (cell hashing), CITE-seq can be used to sequence antibody-bound tags alongside the cellular mRNA, thus reducing costs of scRNA-seq by performing it at the same time on multiple barcoded samples in a single run. Here, we illustrate an ideal CITE-seq data analysis workflow by characterizing the transcriptome of SH-SY5Y neuroblastoma cell line, a widely used model to study neuronal function and differentiation. We obtained transcriptomes from a total of 2879 single cells, measuring an average of 1600 genes/cell. Along with standard scRNA-seq data handling procedures, such as quality checks and cell filtering procedures, we performed exploratory analyses to identify most stable genes to be possibly used as reference housekeeping genes in qPCR experiments. We also illustrate how to use some popular R packages to investigate cell heterogeneity in scRNA-seq data, namely Seurat, Monocle, and slalom. Both the CITE-seq dataset and the code used to analyze it are freely shared and fully reusable for future research.

## 1. Introduction

Single-cell studies are becoming more popular across the field of biomedical research for the level of resolution they can offer, especially in the field of transcriptome analysis. Single-cell RNA-sequencing (scRNA-seq) was first described by Tang et al. in 2009 [1] as a method for transcriptome analysis with higher sensitivity that allowed the study of samples with a very small number of cells. In this study, they focused on the early-phases of embryonic development, specifically the blastomere, a stage in which the embryo is composed by around 30 cells, too few for standard bulk RNA-Seq, which requires hundreds of thousands of cells as starting material [1]. Since then, scRNA-seq has irreversibly transformed the field of cell biology research, by making it possible to capture the complexity of the transcriptome of higher eukaryotes within each cell forming a specific tissue through different developmental stages [2]. In recent years, the improvement of scRNA-seq, together with the development of different technical approaches, made it more affordable and widely used. This technology provides a detailed view of the sample heterogeneity and allows to identify rare cellular clusters that get overlooked by standard methods of bulk RNA-seq [2,3]. Hence, scRNA-seq has been applied across different fields of biomedical research, for instance, to identify and classify new subpopulations of the bone marrow stroma [4], to determine cell-fate decisions and cell trajectories in mouse neural crest [5], and to study the mechanisms of drug-resistance in metastatic bladder cancer by analyzing changes in the response to drugs in both cancer cells and the tumor microenvironment, a highly heterogeneous component of tumors [6].

A number of ingenious and technically different scRNA-seq approaches are currently available, all sharing the same basic steps: cells are separated via enzymatic degradation of the extracellular matrix and subsequently isolated from one another in a process that is accomplished using either cell sorting, microfluidics or microdroplet-based separation [7]. The latter, utilized by the widely used 10X-genomics workflow, divides cells by dispersion of aqueous droplets in an oily stream where each droplet carries a bead with several copies of a barcode. Droplets are more numerous than cells, so ideally, the vast majority of droplets will contain either zero or one cells. Within the droplet, each cell is lysed, and mRNAs are captured by oligo-dT primers with specific nucleotide barcodes that are unique for each droplet. The oligo-dT primers are designed to remove ribosomal RNA from subsequent analyses, which constitutes ~80% of all RNA in the cell [8]. A reverse transcription reaction then occurs, producing cDNAs that will contain the reverse complement of the mRNAs, preceded by the specific cell barcode sequence. Following steps of cDNA preparation for sequencing will then produce abundance estimates of each original mRNA with information on the cell of origin [9].

Since the costs of reaction for generating microdroplet cDNAs can be high, a recent technical development to further multiplex multiple samples has been developed, named Cellular Indexing of Transcriptomes and Epitopes by Sequencing (CITE-seq) [10]. CITE-seq is a multimodal single cell analysis technique which combines the immunophenotyping capabilities of cell sorting with the single cell resolution of scRNA-seq. Antibodies against cell surface markers are conjugated to a nucleotide sequence, called antibody-derived tag (ADT), which is composed of a PCR handle, a specific antibody barcode, and a polyA tail. These antibody-oligo complexes are incubated with the single cells inside the droplets, so that their polyA tails bind to the oligo-dT sequences of the beads and can be reverse-transcribed at the same time of the mRNA from the cell. The antibody barcode allows then to detect which antibodies were bound to the cell, enabling to compare the results from immunophenotyping and the data from transcriptomic analysis [10]. Recently, CITE-Seq has been improved with a novel feature called Cell Hashing. This method uses hashtag oligonucleotides (HTOs): a pool of antibodies against ubiquitously expressed cell surface markers that is conjugated with a 12-bp nucleotide (HTO), which is specific for each sample and it is used to distinguish each sample after pooling them together. The idea behind Cell Hashing is to sequence antibody tags alongside the cellular mRNA, thus reducing costs of scRNA-seq by performing it at the same time on multiple samples and in a single run (i.e., multiplexing the experiment) using barcoded antibodies [11].

In this paper, we present a bioinformatics workflow to analyze CITE-seq data derived from two separate and untreated flasks of SH-SY5Y cells (also indicated simply as SY5Y). SY5Y is a neuroblastoma-derived subclonal line of SK-N-SH, a cell line that was obtained in 1973 from a metastasis in the bone marrow of a four-year-old female. SY5Y were then derived from a neuroblast-like subclone of SK-N-SH, called SH-SY, which was furtherly subcloned two more times to obtain currently available SY5Y cells [12,13]. Unlike other popular neuroblastoma cell models (e.g., Kelly and BE2C [14]), SY5Y is characterized by a normal copy number of the oncogene MYCN, a F1174L mutation in the ALK gene, and by a number of genomic aberrations such as deletions in the q23 band of chromosome 11 and by amplifications of the q21 band of chromosome 17 [15].

We present here a detailed step-by-step workflow designed to analyze CITE-seq data for transcriptome exploration. At the same time, we will provide an unprecedented characterization of the SY5Y neuroblastoma cell line e.g., by re-evaluating suitable housekeeping genes to be used for qRT PCR experiments. The current protocol can be extended to analyze more complex CITE-seq data, e.g., derived from the sequencing of heterogeneous tissues, organs, or tumors.

## 2. Materials and Methods

### 2.1. Cell Culture

SY5Y neuroblastoma cells were cultured in T-75 flasks (CytoONE^®^, Starlab #CC7682-4175, Milan, Italy) using DMEM high glucose, GlutaMAX™, pyruvate medium (Gibco™, Thermo Fisher Scientific #10569010, Waltham, MA, USA) supplemented with 10% fetal bovine serum (Gibco™, Thermo Fisher Scientific #10270106, Paisley, Scotland, UK) and 1% Penicillin-Streptomycin (Gibco™, Thermo Fisher Scientific #15070063, Waltham, MA, USA), and maintained at 37 °C in a humidified atmosphere with 5% CO_2_. Medium was changed twice a week and cells were passaged for maintenance when 90% confluence was reached in the culture flask.

### 2.2. Cell Hashing and Library Preparation for 10X Genomics Sequencing

Cells reaching > 80% confluence coming from two different culture flasks were harvested for sequencing. Cell hashing was performed following manufacturer’s instructions (TotalSeq™-A Cell Hashing and TotalSeq™-A Antibodies Protocol for Simultaneous Proteomics and Transcriptomics with 10X Single Cell 3’ Reagent Kit v2; BioLegend, San Diego, CA, USA). Briefly, after cells counting and viability assessment, single cells suspension samples were incubated 10 min on ice with Human TruStain FcX™ Fc Blocking reagent (BioLegend). Next, sample-specific TotalSeq A antibodies (TotalSeq-A0251 anti-human hashtag 1 Antibody and TotalSeq-A0252 anti-human Hashtag 2 Antibody, BioLegend) were added with subsequent incubation on ice for 30 min. Cells were washed three times with Cell Staining Buffer (BioLegend, Cat# 420201) and finally resuspended in PBS. Cells were counted in an automatic cell counter (Cell counter Thermo Scientific) and samples pooled together. Primers to cDNA PCR (HTO PCR additive primer) were added to increase yield of hashtag oligonucleotide (HTO, cDNA derived from the TotalSeq™ hashtag antibodies). After cDNA amplification, HTO-derived cDNAs (180 bp) and mRNA-derived cDNAs (>300 bp) 2.0 Fluorometer (Invitrogen^®^, Waltham, MA, USA) and Agilent Bioanalyzer DNA assay (Agilent^®^, Santa Clara, CA, USA). Libraries were prepared for sequencing following manufacturer’s instructions (Illumina^®^, San Diego, CA, USA) and then sequenced in 150 bp paired-end mode on Illumina^®^ NovaSeq6000.

### 2.3. Data Processing and Analysis

Raw reads were mapped to the human genome version hg38/GRCh38 using the STAR aligner version 2.7 [16]. The resulting aligned reads data were saved in BAM format, which also included unaligned reads. This BAM was then processed with Cell Ranger v4.0.0, in order to obtain matrices of gene counts per cell in CSV (comma-separated value) format. The dataset has been deposited to GEO under the following accession: GSE171391.

Gene count matrices were loaded in the R statistical software version 4.0.4 running Bioconductor version 3.12. Where not differently indicated, all plots were produced by using base R functions or ggplot2 package version 3.3.3. Data log normalization and scaling was performed using the Seurat package version 4.0.0 [17], which was also used for data dimensionality reduction, cell-cycle regression analysis, and differential expression analysis among conditions. Assignment of cells to cell cycle phases was performed using the cell cycle genes defined in [18,19]. Cluster and state trajectory analysis was performed with the Monocle3 package version 0.2.3.0 [20], and single-cell heterogeneity investigated by factorial single-cell latent variable model implementation in slalom (version 1.12.0) [21] using WikiPathways gene annotations [22]. The corto package version 1.1.4 [23] was used to infer a SY5Y specific co-expression network and Cytoscape was used for network visualization [24]. All code to reproduce the analysis is given in a fully reproducible R Markdown document in Supplementary Material. Alternatively, it can be accessed on GitHub at the following address: github.com/N0toriou5/CITE-seq_pipeline (accessed on 9 April 2021).

## 3. Results

### 3.1. Quality Check and Cell Selection

**Figure 1:**
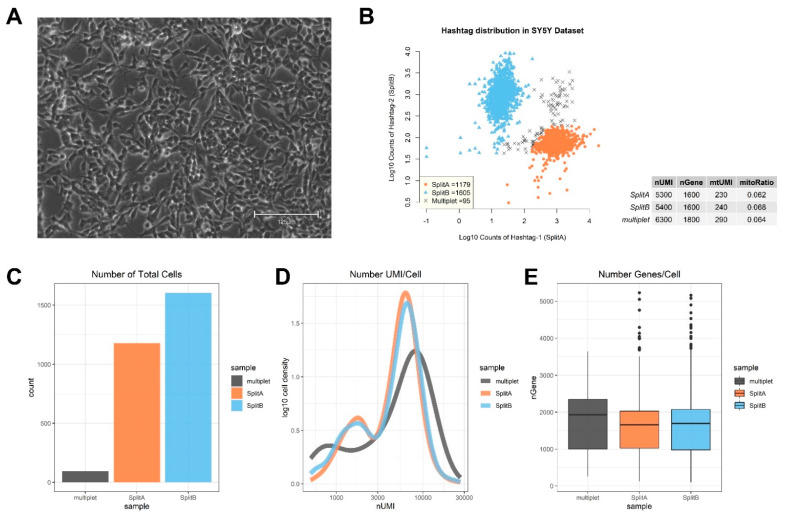
Exploratory analysis of single-cell mRNA expression on SY5Y cell line. (**A**) SY5Y cells were left growing in flask until reaching about 90% of confluence before splitting to two flasks for library preparation and sequencing. (**B**) Scatterplot showing Log10 counts of HTOs. Three populations are distinguished on the basis of Hashtag counts: cells belonging to SplitA, cells belonging to SplitB, and multiplets. Proportions of UMI counts, measured genes, mtUMI and mitoRatio are reported in the table, and QC plots summarizing these metrics are shown in (**C**) barplot, showing the number of cells assigned to each sampling group, (**D**) density plot showing a similar number of UMI/cell in both SplitA and B, while multiplets contained higher UMI/cell, and (**E**) Box and Whisker plots showing the number of genes detected per cell in the three groups.

Cells reaching 90% confluence (Figure 1A) were split into two culture flasks (tagged as SplitA and SplitB) and left growing until reaching > 80% confluence before sequencing. Cells were not synchronized during cell culture. A raw counts sparse matrix in .h5 format, generated by the 10X Genomics software Cell Ranger, was converted to a comma-separated value file (.csv) and imported in R for further analysis. The information on hashtag counting derived from the CITE-seq experiment, hashtag 1 and 2, is already present in this raw counts matrix, as the two hashtags were counted alongside other genes, on a cell-by-cell basis.

We used the hashtag information to assign the cells to the correct sample, namely SplitA and SplitB, on the basis of hashtag counts (Figure 1B). We chose an arbitrary threshold (in our case, threshold was ≈2.3) and a ratio above 3 to assign cells to one split, or the other, while assigning all cells with ambiguous hashtag assignation to the “multiplet” group. A multiplet is the result from two different cells being tagged by the same bead barcode, making them indistinguishable from one another and not suitable for further analysis (specific software has been specifically designed to identify potential multiplets in scRNA-seq data, such as DoubletDecon [25] and Scrublet [26]). The presence of the antibody barcodes makes it easier to identify multiplets, thus excluding this potential source of bias from downstream analysis. Out of a total of 2879 single cells mapped, 1179 were uniquely assigned to SplitA, 1605 to SplitB and 95 tagged as multiplets (Figure 1B,C). Quality check (QC) analysis is given in Figure 1C–E. Briefly, a similar mean amount of Unique Molecular Identifiers (UMI), corresponding to uniquely mapped reads, was detected in both SplitA and B (5300 and 5400, respectively), while a higher amount was observed in multiplets (6300). A mean of 1600 genes/cell were measured in both splits. Mitochondrial genes were similarly detectable in both splits (mtUMI). The mean mitochondrial genes proportion (mitoRatio), i.e., the fraction of mitochondrial transcript counts out of the total transcript counts, was lower than 1% in all the three groups. The mitoRatio is used as an index to identify apoptotic, stressed, or low-quality cells in the data, since these cells show higher proportion of mitochondrial transcripts compared to total. Usually, cells having a mitoRatio higher than 5% are considered as low quality samples and excluded from analysis, but appropriate thresholds can be established during exploratory data analysis.

Raw counts matrix was then filtered to exclude cells expressing less than 500 UMI, 300 genes, and showing a mitochondrial genes proportion of more than 15%. We then took advantage of Seurat function CreateSeuratObject to further refine our dataset by keeping only cells where at least 1000 genes were measured by considering all the genes detected in at least three cells. Standard Seurat LogNormalize method was applied to obtain an expression matrix as follows: data are multiplied by a scale factor (default is 10,000) and log-transformed. The Seurat object is a convenient way to store both expression data (e.g., the count matrix, log normalized expression matrix) and analysis steps (such as dimensionality reduction, or clustering results) for a single-cell dataset.

### 3.2. Global Gene Expression Analysis

**Figure 2:**
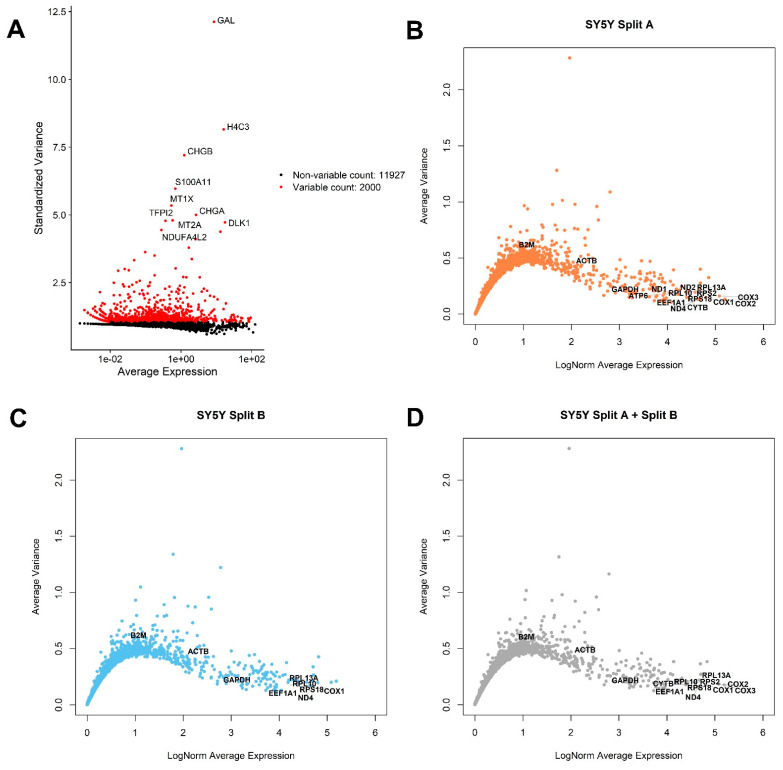
Exploratory analysis of top varying and most stable genes across the dataset. (**A**) Seurat standardized variance vs. average expression plot. The top 10 most varying genes are indicated. Most average expressed genes (*x*-axis) with lowest average variance (*y*-axis) are indicated in (**B**) SplitA, (**C**) SplitB, and (**D**) in the entire dataset. Genes showing an average variance <0.2 and >4 LogNorm Average Expression are labeled, together with commonly used HK genes, such as ACTB, B2M, and GAPDH.

A common first step into single-cell RNA-seq analysis is to identify genes showing a high cell-to-cell expression variation, which are usually those genes better explaining the complexity of the dataset. The Top 2000 variable genes are highlighted in Figure 2A by using default FindVariableFeatures Seurat function. Among variable genes, the ten most variable were GAL, H4C3, CHGB, S100A11, MT1X, TFPI2, CHGA, MT2A, DLK1, NDUFA4L2. In a previous report of our laboratory, phenotyping of MYCN-amplified Kelly and SK-N-BE-2-C neuroblastoma cell lines, a high expression variance of metallothionein genes, such as MT2A and MT1X, was already reported, especially in Kelly cells [14].

**Figure 3:**
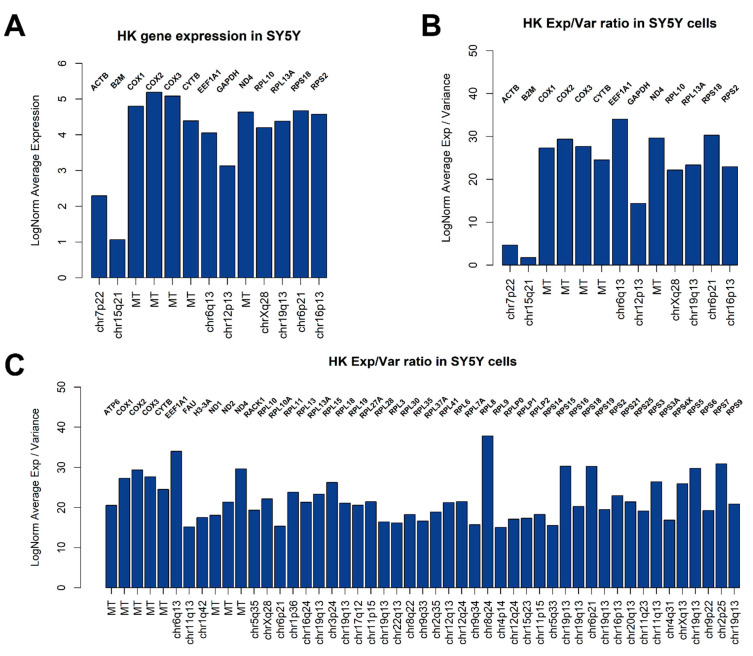
Candidate SY5Y HK genes. Bar plots showing 13 genes selected in Figure 2D compared for LogNorm average expression (**A**) or LogNorm Average Expression/Variance Ratio (**B**). Using ratios seems to be a better choice to identify most stable genes suitable as HK. In (**C**), all genes showing a ratio > 15 are indicated. Forty-nine genes are suitable HK candidates.

An open and frequent debate is how to select proper genes for qPCR analysis normalization (i.e., Housekeeping Genes, HK). Generally speaking, to be considered as a good HK, a gene should be highly expressed in all cells, showing a very low variance. We therefore applied these criteria to identify potential HK genes for SY5Y cells, also checking the expression of commonly used HK genes for qPCR normalization in neuroblastoma experiments involving cell lines, such as ACTB, B2M, and GAPDH (Figure 2B–D). We applied these criteria to identify potential HK genes for SY5Y cells. As showed in Figure 2B,C, a substantial similar expression of B2M, ACTB, GAPDH, EEF1A1, RPL13A, RPL10, ND4, RPS18, and COX1 can be observed when analyzing the cells from different flasks separately, while 13 potential HK genes emerge when considering the entire dataset, (Figure 2D), summarized for expression and geographic location on human genome in Figure 3A. We also investigated the presence of potentially interesting HK candidates not only considering absolute expression and variance thresholds for subsetting genes, but also considering the ratio between average LogNorm Exp on Variance, since high ratios may indicate good HK candidates. As showed in Figure 3B, while commonly used for qPCR data normalization, both ACTB and B2M showed the lowest ratios among 13 HK candidates, thus being poorly suitable as expression normalizers. We therefore extended our search including all genes showing an average LogNorm Exp/Var ratio higher than 15. In Figure 3C, 49 genes were selected. Most of the candidate HKs in Figure 3C code for ribosomal proteins (39), while some of them (8) are mitochondrial genes. Among classically used HK genes for qPCR, such as ACTB and B2M, GAPDH seems the best choice, showing a strong expression/variance ratio. Other interesting non-ribosomal, non-mitochondrial genes emerge, such as EEF1A1 (Eukaryotic Translation Elongation Factor 1 Alpha 1) and H3-3A (H3.3 Histone A). Both EEF1A1 and H3-3A have been previously described as good HK genes [27,28].

### 3.3. Dataset Dimensionality Reduction and Cell-Cycle Regression

**Figure 4:**
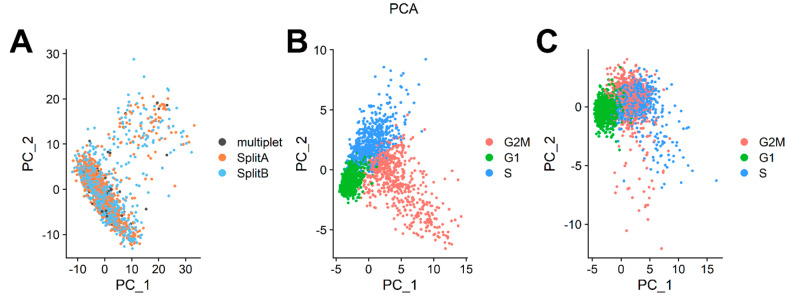
Principal Component Analysis. (**A**) Single cells are plotted along the first two components retaining the highest variance. No difference among the two splits, or multiplets, was detectable. (**B**) Principal component analysis on cell cycle genes showed a clear separation of cells according to cell cycle genes expression. (**C**) Partial regression removed the cell cycle variance, maintaining the difference between G1 and G2M/S cells. Partial regression also allowed retaining signals separating non-cycling and cycling cells, while removing differences in cell cycle phases amongst proliferating cells (which are often uninteresting).

We further processed data by applying the data scaling process from Seurat’s ScaleData function, which applies a linear transformation so that highly expressed genes do not dominate downstream analysis. Data scaling is a standard pre-processing step prior to dimensional reduction. Since not all detected genes (13,927 in our case) are meaningful to classify cells into biologically relevant clusters based on their expression profiles, dimensionality reduction techniques are employed to reduce data complexity and for data visualization. Furthermore, computational requirements for several downstream analysis techniques (e.g., cell clustering) benefit from dimensionality reduction, because several clustering and classification algorithms require large computational resources to analyze the original dataset space. Principal Component Analysis (PCA) is a very popular technique to reduce the original space in few meaningful dimensions. PCA performs an orthogonal transformation of the original dataset to create a set of new, uncorrelated variables (i.e., the principal components). These principal components are linear combinations of variables in the original dataset, ranked in decreasing order of variance. In Figure 4A, cells are plotted along the first two principal components of the dataset. No difference between cells belonging to SplitA, SplitB, or multiplet group was detectable. We determined which PC exhibited cumulative percent greater than 90% and a percentage of variation associated with the PC as less than 5. Twelve PCs were enough to capture more than 90% of the complexity of the dataset.

We expected that a cell line would show very little genomic variability with most of the differences in transcriptome that may be explained by cell cycle phase, which is a common source of variability in asynchronous cell cultures. Seurat contains a set of useful functions to assign a cell cycle score to cells on the basis of cell cycle markers defined by [18]. As showed in PCA in Figure 4B, cells tend to occupy the plot space according to cell cycle phase. To mitigate the effect of cell cycle phase and to investigate transcriptome differences among cells relying on, for example, differentiating processes, proliferation rates or aggressiveness behavior, we regressed out the difference between the G2M and S phase scores by ScaleData function to keep the source of transcriptome variability separating non-cycling and cycling cells, while regressing out differences in cell cycle phase among proliferating cells. As showed in Figure 4C, partial cycle regression resulted in a clear separation of cells belonging to G1, while keeping together cells in G2M/S phases, or actively dividing cells. To note, we also totally regressed out cell cycle scores (Supplementary Figure S1, right panel), but this procedure does not have an impact on downstream analyses. This is because our cell model was sequenced when culturing cells reached a confluence >80%, and for SY5Y cells that grow as a monolayer (Figure 1A) this means a decrease in growth rate. However, we strongly recommend applying partial regression of the cell cycle, particularly when the focus of the analysis is to dissect differentiating processes (e.g., hematopoiesis), where we expect a quiescent, and an actively proliferating population to coexist in culture.

### 3.4. Differential Expression and Cluster Analysis

**Figure 5:**
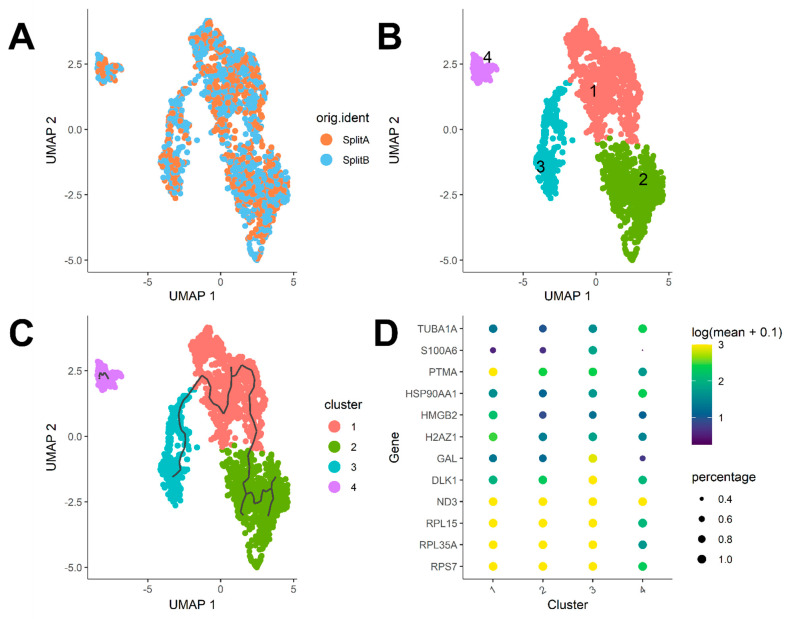
Cluster Analysis. (**A**) UMAP 2D projection of single cells. No detectable difference was observed between splits. (**B**) Cells can be assigned to four cluster-forming communities applying the Leiden algorithm. (**C**) Unsupervised trajectory learning. Most variant genes show expression differences following a path from cluster 3 to 2. Cluster 4 remains separated from the others. (**D**) Top 12 genes characterizing each community. Cluster 4 show the highest expression of TUBA1A and HSP90AA1, while S100A6 was poorly expressed in a minimal fraction of cells (<0.1). Higher PTMA, HMGB2, and H2AZ1 expression characterizes cluster 1.

The Seurat FindMarkers function offers a simple way to test for differentially expressed genes for each of the identity classes in a dataset. In our case, we were interested in understanding if transcriptome differences existed between SplitA and B. As expected, no differentially expressed gene was detectable between the two flasks, as they came from a single split from the same original culture. Thus, to investigate if a source of heterogeneity among cells could be captured, we transferred the cell cycle regressed expression matrix (keeping only cells belonging to SplitA and B) to a Monocle cell data set object using convenient functions from the SeuratWrappers package, which is a collection of wrapper functions to transfer data processed in Seurat to other single-cell analysis packages. After data normalization and applying graph based Uniform Manifold Approximation and Projection (UMAP) dimensionality reduction in monocle, we visualized again the cells in a 2D plot coloring cells by split identity. As showed in Figure 5A, cells from the two splits are distributed uniformly in the reduced space, meaning that no substantial difference came from splitting. However, point distribution was suggestive of different cluster forming groups among cells. We therefore applied the cluster_cells function implemented in Monocle3, choosing a community detection algorithm relying on Leiden clustering approach [29]. Applying a low-resolution threshold (e.g., 10^−3^), we assigned cells to the four cluster-forming communities in Figure 5B. Then, we asked whether differences among communities may be explained by transition from different functional states. Monocle3 offers some functions to investigate transient transcriptomic changes among cell states by learning the sequence of gene expression changes each cell should go through when placed in a dynamic biological process. Once a trajectory of gene expression changes has been built, each cell can be placed at its appropriate position through the trajectory as showed in Figure 5C, where trajectories were traced in an unsupervised manner. When multiple trajectories are equally possible, monocle3 build branches, as showed clearly in cluster 2. Trajectory analysis usually performs very well when different states driven by a defined subset of genes exists, e.g., during hematopoiesis or embryogenesis processes. A similar kind of analysis can be performed by using the spathial R package [30], which takes advantage of an unsupervised learning method to navigate cells’ features to identify transition states among communities. This is a slightly different approach from monocle, because spathial is able to reconstruct transcriptome states that explain possible transcriptomes, which are representative of real intermediate states between cells belonging to different clusters.

Differential analysis can be performed through the top_markers function to identify genes most characterizing each cell state through the trajectory. As showed in Figure 5D, major sources of variation are detectable among cluster 4 and the other three clusters, as cluster 4 showed a higher expression of TUBA1A (Tubulin Alpha 1a) and HSP90AA1 (Heat Shock Protein 90 Alpha Family Class A Member 1), while expression of S100A6 (S100 Calcium Binding Protein A6) was barely detectable in a minimal fraction of cells. Higher PTMA (Prothymosin Alpha), HMGB2 (High Mobility Group Box 2), and H2AZ1 (H2A.Z Variant Histone 1) expression characterized cluster 1, while cluster 2 and 3 were substantially homogeneous, despite a marked difference in GAL (galanin and GMAP pre-propeptide) and DLK1 (delta like non-canonical notch ligand 1) expression, mostly characterizing cluster 3.

### 3.5. Dissection of Cell Heterogeneity by Factorial Single-Cell Latent Variable Model Method

Factorial single-cell latent variable model (f-scLVM) is a method to investigate sources of heterogeneity in scRNA-seq datasets by using pathway annotations to drive the inference of interpretable factors explaining variability among cells, facilitating the discovery of meaningful subpopulations [21]. We applied the f-scLVM method implementation in the R package slalom to analyze our data, coupled with human gene annotations deposited in WikiPathways [22] to define annotated terms of heterogeneity.

**Figure 6:**
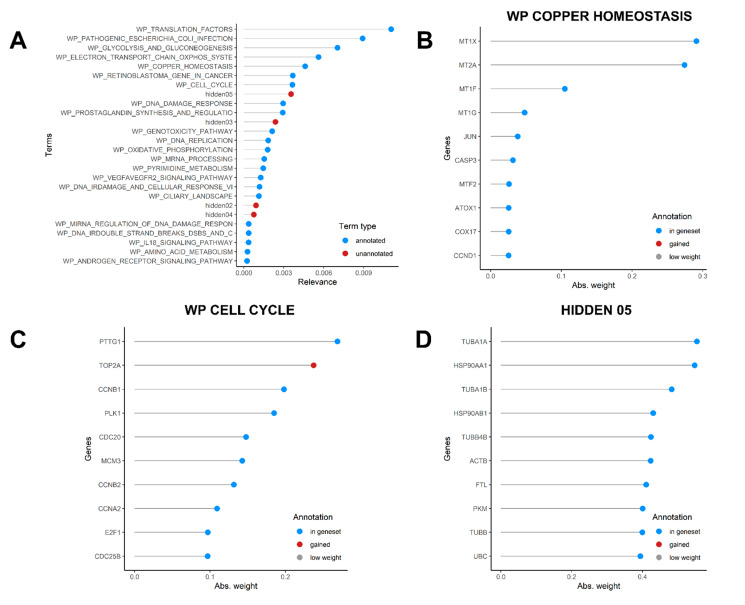
Slalom output. (**A**) Graph showing the most relevant factors identified by the f-scLVM model, both annotated in Wiki Pathways (blue) or not annotated (red) (**B**) Most relevant genes in the WP COPPER HOMEOSTASIS component (**C**) Most relevant genes in the WP CELL CYCLE component, (**D**) Most relevant genes in the hidden05 component.

Figure 6A shows top pathways explaining the heterogeneity in the dataset. The highest relevance pathway was the WP TRANSLATION FACTORS, whose top relevant genes were several elongation factors responsible for the enzymatic delivery of aminoacyl tRNAs to the ribosome (Supplementary Figure S2), including EEF1A1. Among top 10 pathways, the most interesting factors are gene sets related to copper homeostasis (Figure 6B) and cell cycle (Figure 6C). In Figure 6B, several metallothioneins are listed as most varying genes in the dataset, while Figure 6C highlighted enhanced DNA Topoisomerase II Alpha expression in some cells, an enzyme involved in DNA transcription. Since we run the model on the original dataset (i.e., before cell cycle score regression), this may evidence that a small fraction of actively dividing cells was present in our dataset (as anticipated in Figure 4B). However, running the monocle pipeline on the same original dataset gave exactly the same results as the regressed dataset analysis (Supplementary Figure S3), meaning that cycling cells could explain the dataset heterogeneity only in minimal part, while major differences depended on genes, such as TUBA1A and HSP90AA1, which were found to characterize cluster 4 (Figure 5D), and the unannotated hidden factor 05 (Figure 6D).

While providing a simple way to analyze variability in scRNA-seq datasets with useful integrated plotting functions, the slalom analysis may take a lot of time to be performed, depending both on the number of pathways and genes that are given in input, and the size of the dataset. In our case, following the standard slalom pipeline as indicated in the package vignette, setting a range for the number of interactions between 1000 and 10,000, and considering 211 annotated factors and 4266 genes, it took almost 6 days to finish the analysis on R v4.0.4, running on a 64 Bit Windows 10 Pro (v20H2) system with an Intel^®^ Core™ i7-9600 CPU @ 3.00 GHz and 64.0 GB RAM. Our model converged after 3050 iterations. We therefore suggest to carefully evaluate and consider performing this kind of analysis on a powerful workstation, or a computational cluster. Furthermore, we strongly recommend saving the results of the analysis to a SingleCellExperiment object as explained in the package vignette, as saving the model in .rData format may incur to data loss.

### 3.6. Use of Single-Cell Data for Co-Expression Network Inference

Gene regulatory networks (GRNs) are representations of gene-gene relationships in a given phenotypic state [31]. Usually, GRNs summarize relationships between transcription factors and target genes, and have become a very popular way to describe disease specific signatures or drug responses [32]. Most common GRNs inference methods rely on co-expression, such as ARACNe, CorTo, and many others [23,31]. Such networks can be used to investigate network rewiring following drug treatments, or to infer the so-called Master Regulators, i.e., Transcription Factors (TFs) driving a particular phenotypic state. It was described that more than 200 gene-expression profiles were required to assemble and analyze regulatory networks [33], consistent with the requirement of ~100 expression profiles for Mutual Information-based analyses [34]. Single-cell RNA-seq are an excellent source of individual samples to build context-specific GRNs. Here, we used the SY5Y single-cell transcriptomes (n = 2143 cells) to build a TF-Target network with the corto algorithm. The network was saved into a regulon object, whose information about TF-Target relationships was exported in tabular form to be loaded in Cytoscape for network visualization (Supplementary Figure S4). A focus of the E2F1 sub-network, which is a key regulator of cell cycle emerged as a source of heterogeneity in Figure 6C, is given as Supplementary Figure S5). The “regulon” object, containing all SY5Y specific TF-Target relationships, is given as supplementary file.

## 4. Discussion

We produced an original untreated SY5Y cell line CITE-seq dataset to illustrate a standard workflow using basic R functions and very popular R packages to analyze single-cell transcriptomic data. SY5Y cells are usually used as a neuronal function and differentiation model. Considering that neuroblastoma is a highly heterogeneous type of cancer, with different stages that largely vary between each other, it is important to identify both major sources of variability among cells and also the most stable genes that can serve as reliable housekeeping genes, since they are highly relevant as expression controls and for normalization purposes when doing differential expression analysis, especially in qRT PCR analysis. Previous studies with qPCR have found HPRT1 and SDHA to show low expression variability in primary neuroblastoma [35] and also, specifically for the SY5Y line, GAPDH, M-RIP, and POLR2F were found to be reliable genes to be used as normalization factors [36]. Our analysis showed that most widely used HK genes for qPCR analysis normalization, such as ACTB and B2M, may not be the best HK genes suitable in experiments involving SY5Y cells, while confirming GAPDH as an optimal HK gene. We also identified a set of 49 genes that are expressed at high levels among SY5Y cells showing a very low variance (thus, maximizing the Expression/Variance ratio). Most of these genes encode for ribosomal or mitochondrial proteins, while some, such as EEF1A1 and GAPDH, encode cytoplasmic genes. EEF1A1 was previously reported as a good HK in bone marrow derived mesenchymal stem cells [27], while suitability of GAPDH was questioned. Our 49 HK candidates selection may be useful for wet lab scientists in need of transcriptionally stable genes when planning qRT-PCR experiments involving SY5Y as cellular model. On the other hand, a major source of variation is given by some genes encoding for metallothionein proteins such as MT2A and MT1X. A high variance in metallothioneins expression was already detected in neuroblastoma cell lines [14], and this could reflect different metabolic states among cultured cells. Neuroblastoma has been demonstrated to be a highly sensitive tumor to heavy metal ion fluctuations, such as zinc, iron, and copper, and to hypoxic conditions or oxidative stresses, all representing stimuli able to induce metallothioneins transcription [37]. Furthermore, as highlighted in Figure 6B, copper homeostasis is a critical source of heterogeneity in SY5Y and neuroblastoma in general [38], thus influencing metallothioneins expression. This variability can be possibly explained by local regions of hypoxia-induced oxidative stresses in the 2D monolayer flask environment, caused for example by the formation of densely populated and hypoxic areas.

The two samples came from two separate flasks derived from the same split, and were kept in homogenous conditions until the sequencing process started; it was therefore reassuring that no significant transcriptional difference could be detected between the two SY5Y biological replicates. A similar single-cell experimental setup, comparing two different neuroblastoma cell lines, highlighted thousands of differentially expressed genes [14]. This shows that the CITE-seq + 10X Genomics procedure, and the subsequent analysis pipeline described here, possesses a negligible false discovery rate for differential expression, and that SY5Y cell line transcriptome is highly constant, making SY5Y a very strong experimental model.

Within both samples, we could however find clusters of cells representing distinct transcriptomic states, partially overlapping with different cell cycle phases (Figure 4C and Figure 5B). The genes characterizing these clusters are involved in neuroblastoma differentiation (TUBA1A, DLK1) [39,40], survival/tumor growth/proliferation (S100A6, HSP90AA1) [41,42], or MYCN expression (PTMA) [43]. Among genes characterizing different cell communities, we also found some ribosomal proteins (RPL15, RPL35A, RPS7), and the mitochondrial gene ND3. Our data showed that RPS7 and RPL15 are highly expressed genes with very low variance throughout the SY5Y dataset, which makes these genes possible HK candidates for this cell line. However, cluster 4 (Figure 5B) is characterized by low levels of these ribosomal proteins but higher levels of TUBA1A, HSP90AA1, and PTMA. While this difference is very little, it can be due to a little population subset characterized by a more aggressive/stressed transient behavior, which can be determined by some stresses casually occurring during 2D culture conditions, rather than a true difference among cells. Several single-cell designed methods, such as Seurat, Monocle, single-cell latent variable model (scLVM) from slalom, or spathial can help dissecting some source of heterogeneity still present even in commonly used tumor cell line models. In particular, the slalom model indicates pathways linked to copper metabolism as a major source of heterogeneity, consistently with the variability in metallothionein levels observed [14,38,44].

A typical single-cell experiment measures thousands of transcripts in thousands of cells. This can be exploited to meet sample size requirements for GRN inference by co-expression-based methods, as we performed using the corto package [23].

In our paper, we illustrated an example workflow for CITE-seq data analysis including cutting-edge methods that can be adapted in several manners to analyze different experimental settings. To this end, fully reproducible and re-usable code is given as Supplementary Material to provide a guide for CITE-seq data analysis. Our dataset on SY5Y confirmed that this cell line is transcriptionally homogeneous and suggested 49 potential candidates to be used as good HK genes for qPCR normalization, with GAPDH or EEF1A1 as best choice to this purpose. A small source of heterogeneity can be identified by in-depth data analysis, possibly caused by fluctuations in genes involved in response to hypoxic stress, metal responses, differentiation, survival, or tumor staging in neuroblastoma, identifying cell communities that adapt to the local environment (e.g., a local increase of cell density) by modulating their transcriptome to alter their metabolism/proliferation rates.

## 5. Conclusions

In conclusion, our paper provides a clear reference guide for CITE-seq data analysis, while re-evaluating the commonly used housekeeping genes and proposing a new set of 49 specific, single-cell tested candidate HK genes for experiments involving SY5Y cells. No significant difference in transcripts levels can be attributed to the splitting of a cell culture. We identified however, even within the homogeneity of the SY5Y cell line, distinct populations independent from cell cycle phases, and characterized by different stress and metabolic states. These subpopulations were found consistently in both biological replicates.

Currently, huge efforts are being undertaken by the Human Cell Atlas [45] and similar projects [46,47] in characterizing the complexity of the human Transcriptome at the single-cell level in living tissues. We also believe that a deep characterization at single-cell resolution of human cell lines will allow for a better usage of these omnipresent models in molecular/cell biology and pharmacology. Many single-cell datasets measured on cell lines exist beyond the one described here (SY5Y cells) on other cell lines from neuroblastoma [14], breast cancer [48], and lymphoblasts [49,50]. Fueled by current technological advances provided by, e.g., CITE-seq, cell line single-cell datasets can be produced in even higher numbers and for more experimental contexts, towards the generation of an integrated and comprehensive Human Cell Line Atlas.

## Data Availability

The raw data associated with this dataset are stored at the Gene Expression Omnibus under the following accession: GSE171391.

## Supplementary Materials

The following are available online at https://www.mdpi.com/article/10.3390/mps4020028/s1, Figure S1, Principal Component Analysis, Figure S2, Monocle Cluster Analysis, Figure S3, Relevant genes in the WP TRANSLATION FACTORS component, Figure S4, SY5Y co-expression network, Figure S5, E2F1 sub-network.

## Abbreviations

ADT	Antibody-Derived Tag
CITE-seq	Cellular Indexing of Transcriptomes and Epitopes by Sequencing
CSV	Comma-Separated Value
GRN	Gene Regulatory Network
HK	Housekeeping Gene
HTO	Hashtag Oligonucleotide
PCA	Principal Component Analysis
QC	Quality Check
scRNA-seq	Single-cell RNA-sequencing
TF	Transcription Factor
UMAP	Uniform Manifold Approximation and Projection
UMI	Unique Molecular Identifier
