# Supplementary material for: The Transcriptome of SH-SY5Y at Single-Cell Resolution: A CITE-Seq Data Analysis Workflow
Source: Methods Protoc. 2021 May 6;4(2):28. doi: 10.3390/mps4020028 (PMC8163004; doi:10.3390/mps4020028)
Supplement: Supplementary file 1 [file mps-04-00028-s001.zip › Supplementary Figures.pdf]

Supplementary Figures

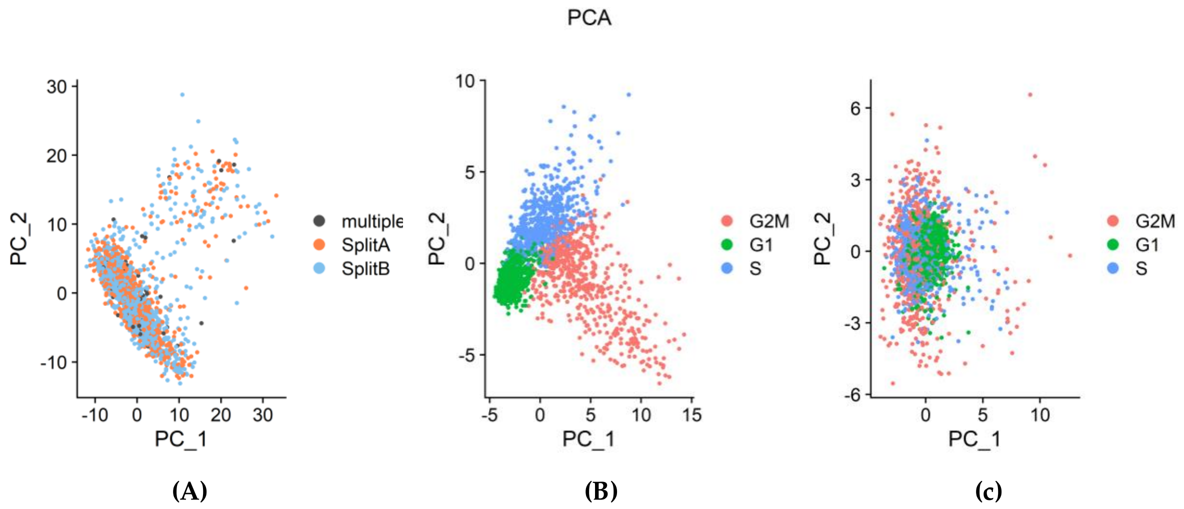

Figure S1. Principal Component Analysis**.** (**A**) Single cells are plotted along the first two components retaining the highest variance. No difference among the two splits, or multiplets, was detectable. (**B**) Principal component analysis on cell cycle genes showed a clear separation of cells according to cell cycle genes expression. (**C**) Complete cell cycle score regression removed the cell cycle variance.

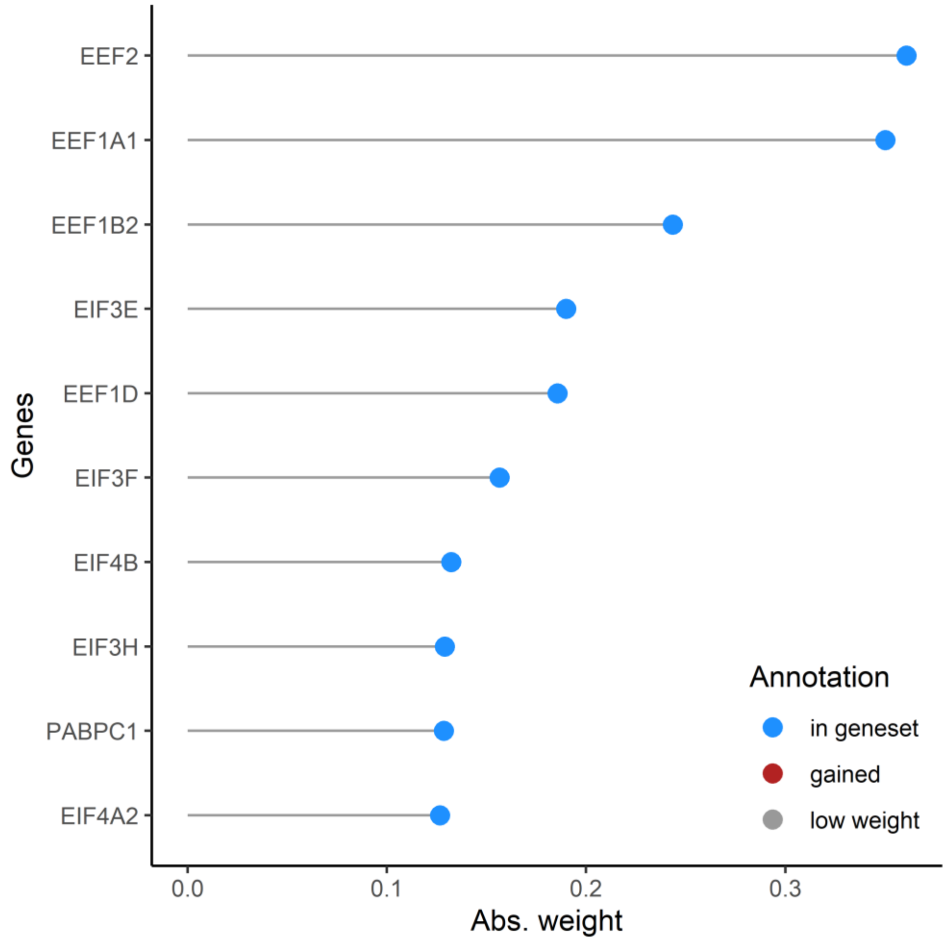

Figure S2. Slalom output. Most relevant genes in the WP TRANSLATION FACTORS component.

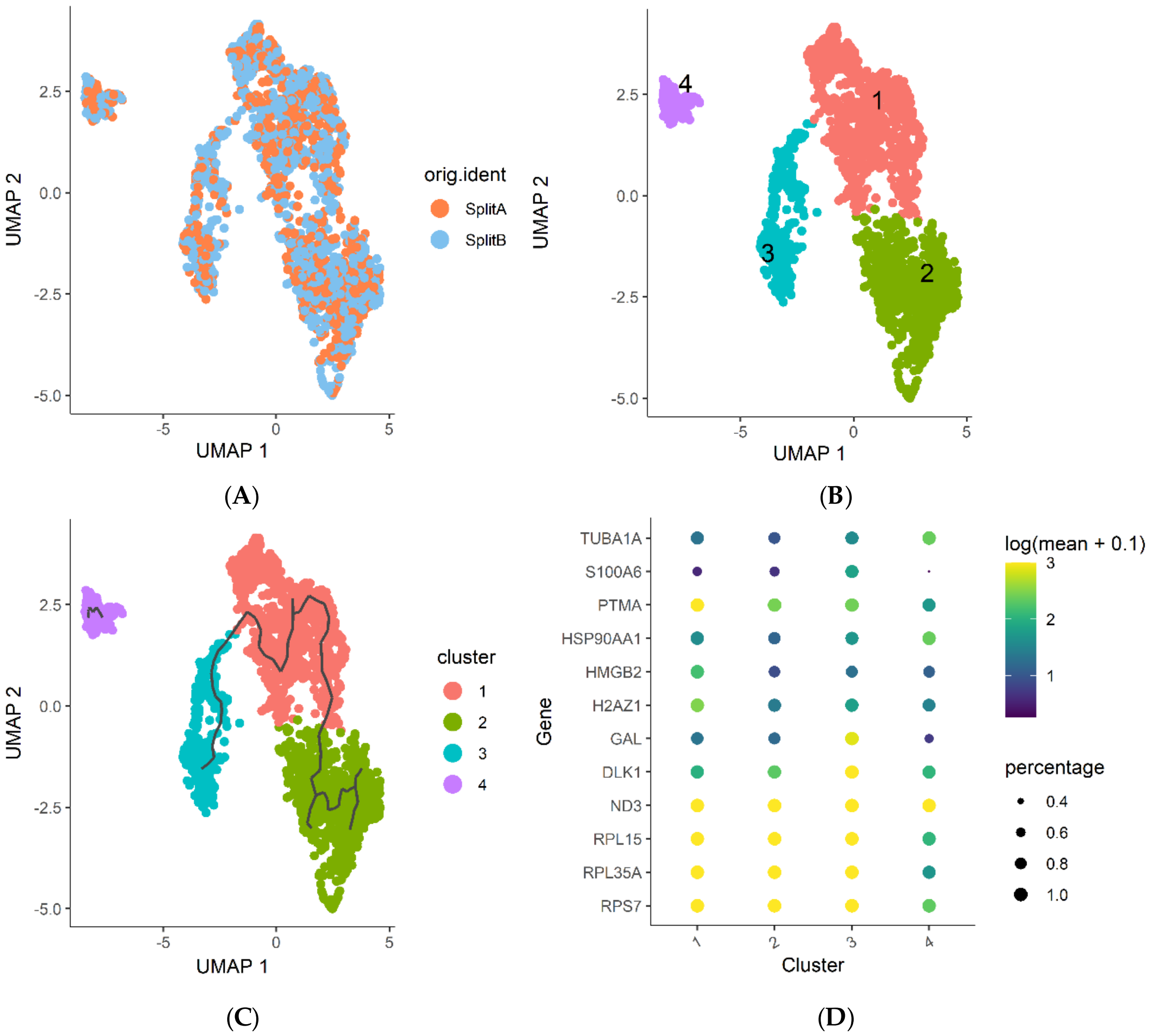

Figure S3. Cluster Analysis. (**A**) UMAP 2D projection of single cells. No detectable difference was observed between splits. (**B**) Cells can be assigned to 4 cluster-forming communities applying the Leiden algorithm. (**C**) Unsupervised trajectory learning. Most variant genes show expression differences following a path from cluster 3 to 2. Cluster 4 remains separated from the others. **(D)** Top 12 genes characterizing each community. Cluster 4 show highest expression of TUBA1A and HSP90AA1, while S100A6 was poorly expressed in a minimal fraction of cells (< 0.1). Higher PTMA, HMGB2 and H2AZ1 expression characterizes cluster 1.

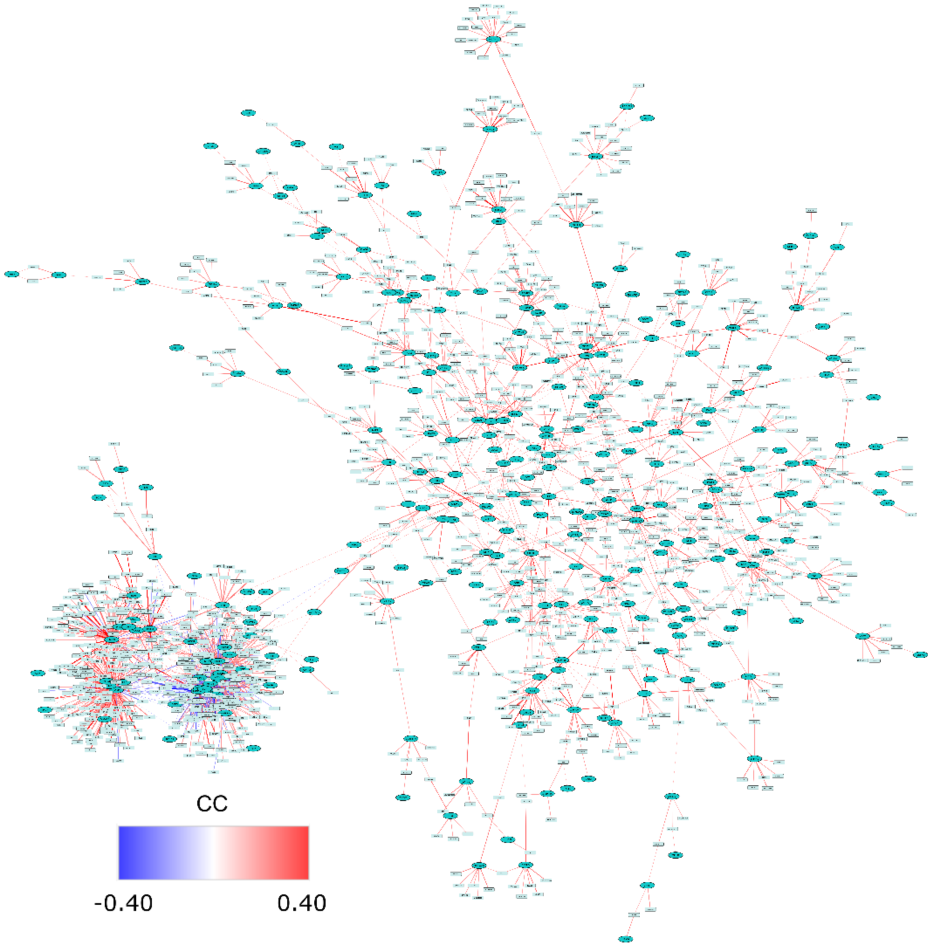

Figure S4. SY5Y co-expression network. TFs nodes are showed as cyan colored ellipses, while targets as light grey rectangles. Thickness of edges is proportional to TF-target measured likelihood, while color scale is proportional to the correlation coefficients between the target and the TF.

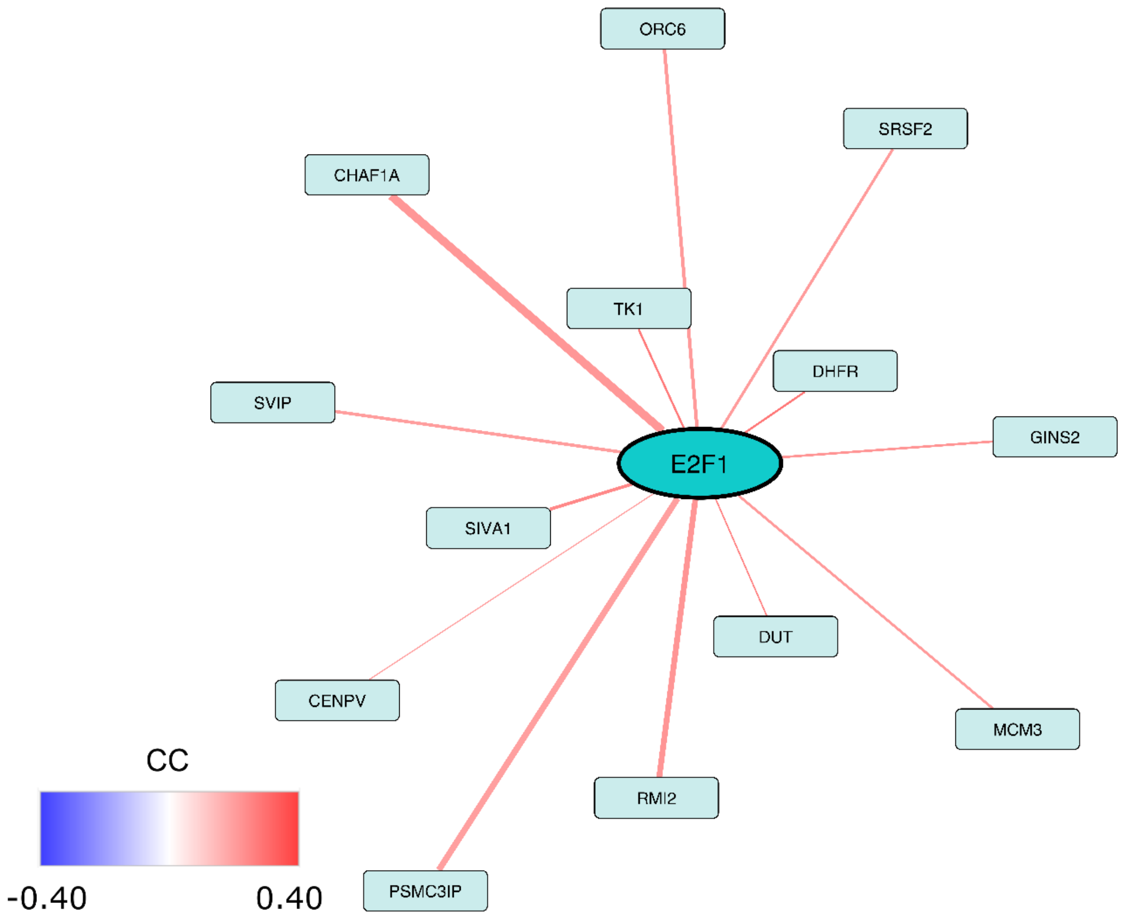

Figure S5. E2F1 sub-network. The E2F1 TF is showed as a cyan colored ellipse, while targets as light grey rectangles. Thickness of edges is proportional to TF-target estimated likelihood, while color scale is proportional to the correlation coefficients between the target and the TF.
