# Supplementary material for: The Transcriptome of SH-SY5Y at Single-Cell Resolution: A CITE-Seq Data Analysis Workflow
Source: Methods Protoc. 2021 May 6;4(2):28. doi: 10.3390/mps4020028 (PMC8163004; doi:10.3390/mps4020028)
Supplement: Supplementary file 1 [file mps-04-00028-s001.zip › rmarkdown/rmarkdown.html]

The transcriptome of SH-SY5Y at single-cell resolution: a CITE-Seq data analysis workflow


# The transcriptome of SH-SY5Y at single-cell resolution: a CITE-Seq data analysis workflow

#### Daniele Mercatelli

#### 07 April 2021

- 1 Extract gene count matrices
- 2 Setting up the R environment for the analysis
- 3 Prepare data
  - 3.1 Identify samples by Hashtags
  - 3.2 Convert gene ids in raw counts data to gene symbols
  - 3.3 QC
  - 3.4 QC Metrics and low quality cells filtering
- 4 FPKM and TPM Normalization, Create Seurat Object
- 5 Explorative Analysis
  - 5.1 Descriptive Plots
  - 5.2 HK genes plot in SY5Y
  - 5.3 Dimensionality reduction and cell cycle score regression
- 6 Monocle Clustering and Analysis
- 7 Building a co-expression network
- 8 Technical Session Info

# 1 Extract gene count matrices

Gene counts were extracted from 10X genomics H5 matrices using cellranger v4.0.0 on Linux Debian 10.8. This yielded comma-separated value files (CSV) with genes as rows, samples (cells) as columns, and values corresponding to the raw read counts observed for every gene/cell combination. The CSV file contained also two rows indicating different hashtag counts. The CSV files are then zipped.

```
# Linux code
sample="SY5Y"
export PATH=/home/notorious/programs/cellranger-4.0.0:$PATH
cellranger mat2csv ${sample}/filtered_feature_bc_matrix.h5 ${sample}.csv
gzip *csv
```

# 2 Setting up the R environment for the analysis

We will load all the packages required by the full analysis, plus extra functions available in the “code/” subdirectory. If the packages are not available, we will install them using Bioconductor. Some packages are available through GitHub.

```
if (!requireNamespace("BiocManager",quietly=TRUE)){
  install.packages("BiocManager")
}
packages<-c("Seurat","ggplot2","cowplot","dplyr","corto","msigdbr","gridExtra","AnnotationHub","GenomicFeatures","devtools","SingleCellExperiment","slalom","ggforce"
)
for(p in packages){
  if (!p %in% rownames(installed.packages())){
    BiocManager::install(p)
  }
  library(p,character.only=TRUE)
}
# if monocle3 and SeuratWrappers are not installed, it is required to install them through install_github function from the "devtools" package
gits<-c("monocle3","SeuratWrappers")
for(g in gits){
  if (!g %in% rownames(installed.packages())){
    library(devtools)
    remotes::install_github('satijalab/seurat-wrappers')
    devtools::install_github('cole-trapnell-lab/leidenbase')
    devtools::install_github('cole-trapnell-lab/monocle3')
  }
  library(g,character.only=TRUE)
}
source("geneids.R") # Functions to convert gene ids
source("expmerger.R") # Functions to sum multiple expression tracks
source("qol.R") # Various "quality of life" functions
source("textplot3.R") # function for labelling samples in plots
library(Seurat)
library(slalom)
library(ggforce)
library(cowplot)
library(dplyr)
library(corto)
library(msigdbr)
library(monocle3)
library(SeuratWrappers)
library(GenomicFeatures)
library(AnnotationHub)
library(gridExtra)
library(SingleCellExperiment)
dir.create("markdown")
dir.create("markdown/plots")
dir.create("markdown/results")
dir.create("markdown/data")
```

# 3 Prepare data

Load raw counts matrix from .csv file

```
# create rawcount matrix
rawcounts<-read.csv(gzfile("SY5Y.csv.gz"),as.is=TRUE,row.names=1)
rawcounts<-as.matrix(rawcounts)
dim(rawcounts) # 33540 ENSEMBL genes, 2879 samples
```

```
## [1] 33540  2879
```

## 3.1 Identify samples by Hashtags

We will assign cells to the correct sample by evaluating hashtag counts. Cells assigned to “multiplet” group will be excluded from further analysis.

```
# Group assignment
x<-rawcounts["Hashtag-1",]
y<-rawcounts["Hashtag-2",]
ll<-185
ratio<-3
hashtags<-setNames(rep(NA,ncol(rawcounts)),colnames(rawcounts))
hashtags[(y<=ll&(x/y>=ratio))|(y==0&x>0)]<-"SplitA"
hashtags[(x<=ll&(y/x>=ratio))|(x==0&y>0)]<-"SplitB"
hashtags[is.na(hashtags)]<-"multiplet"
pchs<-htcols<-hashtags
htcols[hashtags=="SplitA"]<-"sienna1"
htcols[hashtags=="SplitB"]<-"skyblue2"
htcols[hashtags=="multiplet"]<-"gray30"
pchs[hashtags=="SplitA"]<-16
pchs[hashtags=="SplitB"]<-17
pchs[hashtags=="multiplet"]<-4
pchs<-as.numeric(pchs)
x<-log10(x+0.1)
y<-log10(y+0.1)

#png("plots/000_hashtags.png",w=3500,h=3500,res=600)
plot(x,y,xlab="Log10 Counts of Hashtag-1 (SplitA)",ylab="Log10 Counts of Hashtag-2 (SplitB)",frame.plot=FALSE,
     pch=pchs,col=htcols,main="Hashtag distribution in SY5Y Dataset")
legend("bottomleft",pch=c(16,17,4),col=c("sienna1","skyblue2","gray30"),bg="ivory",
       legend=c(
         paste0("SplitA =",table(hashtags)["SplitA"]),
         paste0("SplitB =",table(hashtags)["SplitB"]),
         paste0("Multiplet =",table(hashtags)["multiplet"])
       )
)
```

Figure S1. Hashtag-1 and -2 Log10 Counts

```
#dev.off()
#save(rawcounts,hashtags,file="data/000_rawcounts_ensembl.rda")
```

## 3.2 Convert gene ids in raw counts data to gene symbols

```
if(!file.exists("markdown/data/000_rawcounts_symbols.rda")){
  ## Convert to gene symbols
  # Ensembl ids mapping to the same gene will see their raw counts summed up
  ensgmat<-rawcounts
  tmp<-ens2eg(rownames(ensgmat))
  convlist<-eg2sym(tmp)
  names(convlist)<-names(tmp)
  rawcounts<-squish(ensgmat,convlist=convlist,method="sum",verbose=TRUE)
  dim(rawcounts) # 23281 genes, 2879 samples
  nrnas<-apply(rawcounts,2,sum)
  #save(rawcounts,hashtags,nrnas,convlist, file="data/000_rawcounts_symbols.rda")
} else {load("markdown/data/000_rawcounts_symbols.rda")}
```

## 3.3 QC

Perform standard scRNA-seq quality checks to exclude low quality/noisy cells before starting the analysis.

```
# Make the column names as the cell IDs and the row names as the gene IDs
gene_ids <- rownames(rawcounts)
cell_ids <- colnames(rawcounts)

# QC metrics metadata
# Create metadata containing only the cell IDs
metadata <- data.frame(row.names = cell_ids, cells = cell_ids, stringsAsFactors = F)
# Add number of UMIs for each gene per cell to metadata
metadata$nUMI <- Matrix::colSums(rawcounts)
# Add number of genes detected per cell to metadata
metadata$nGene <- Matrix::colSums(rawcounts > 0)
# Add number of UMIs per gene for each cell to metadata
metadata$log10GenesPerUMI <- log10(metadata$nGene) / log10(metadata$nUMI)
# Add sample name associated with each cell to metadata (we only have one sample, so more important when you have multiple samples)
metadata$sample <- hashtags
# get list of mt-genes
library(AnnotationHub)
# Connect to AnnotationHub
ah <- AnnotationHub() # snapshotDate(): 2020-10-27
# Access the Ensembl database for organism
ahDb <- query(ah, 
              pattern = c("Homo sapiens", "EnsDb"), 
              ignore.case = TRUE)
# Check versions of databases available
ahDb %>% 
  mcols()
```

```
## DataFrame with 18 rows and 15 columns
##                          title dataprovider      species taxonomyid      genome
##                    <character>  <character>  <character>  <integer> <character>
## AH53211 Ensembl 87 EnsDb for..      Ensembl Homo sapiens       9606      GRCh38
## AH53715 Ensembl 88 EnsDb for..      Ensembl Homo sapiens       9606      GRCh38
## AH56681 Ensembl 89 EnsDb for..      Ensembl Homo sapiens       9606      GRCh38
## AH57757 Ensembl 90 EnsDb for..      Ensembl Homo sapiens       9606      GRCh38
## AH60773 Ensembl 91 EnsDb for..      Ensembl Homo sapiens       9606      GRCh38
## ...                        ...          ...          ...        ...         ...
## AH78783 Ensembl 99 EnsDb for..      Ensembl Homo sapiens       9606      GRCh38
## AH79689 Ensembl 100 EnsDb fo..      Ensembl Homo sapiens       9606      GRCh38
## AH83216 Ensembl 101 EnsDb fo..      Ensembl Homo sapiens       9606      GRCh38
## AH89180 Ensembl 102 EnsDb fo..      Ensembl Homo sapiens       9606      GRCh38
## AH89426 Ensembl 103 EnsDb fo..      Ensembl Homo sapiens       9606      GRCh38
##                    description coordinate_1_based             maintainer
##                    <character>          <integer>            <character>
## AH53211 Gene and protein ann..                  1 Johannes Rainer <joh..
## AH53715 Gene and protein ann..                  1 Johannes Rainer <joh..
## AH56681 Gene and protein ann..                  1 Johannes Rainer <joh..
## AH57757 Gene and protein ann..                  1 Johannes Rainer <joh..
## AH60773 Gene and protein ann..                  1 Johannes Rainer <joh..
## ...                        ...                ...                    ...
## AH78783 Gene and protein ann..                  1 Johannes Rainer <joh..
## AH79689 Gene and protein ann..                  1 Johannes Rainer <joh..
## AH83216 Gene and protein ann..                  1 Johannes Rainer <joh..
## AH89180 Gene and protein ann..                  1 Johannes Rainer <joh..
## AH89426 Gene and protein ann..                  1 Johannes Rainer <joh..
##         rdatadateadded preparerclass                                     tags
##            <character>   <character>                                   <list>
## AH53211     2017-02-07      AHEnsDbs                   EnsDb,Ensembl,Gene,...
## AH53715     2017-04-05      AHEnsDbs                   EnsDb,Ensembl,Gene,...
## AH56681     2017-06-29      AHEnsDbs                   EnsDb,Ensembl,Gene,...
## AH57757     2017-08-31      AHEnsDbs                   EnsDb,Ensembl,Gene,...
## AH60773     2017-12-21      AHEnsDbs                   EnsDb,Ensembl,Gene,...
## ...                ...           ...                                      ...
## AH78783     2019-10-29      AHEnsDbs               99,AHEnsDbs,Annotation,...
## AH79689     2020-04-27      AHEnsDbs              100,AHEnsDbs,Annotation,...
## AH83216     2020-04-27      AHEnsDbs              101,AHEnsDbs,Annotation,...
## AH89180     2020-10-27      AHEnsDbs              102,AHEnsDbs,Annotation,...
## AH89426     2020-10-27      AHEnsDbs 103,Annotation,AnnotationHubSoftware,...
##          rdataclass              rdatapath              sourceurl  sourcetype
##         <character>            <character>            <character> <character>
## AH53211       EnsDb AHEnsDbs/v87/EnsDb.H.. http://www.ensembl.org     ensembl
## AH53715       EnsDb AHEnsDbs/v88/EnsDb.H.. http://www.ensembl.org     ensembl
## AH56681       EnsDb AHEnsDbs/v89/EnsDb.H.. http://www.ensembl.org     ensembl
## AH57757       EnsDb AHEnsDbs/v90/EnsDb.H.. http://www.ensembl.org     ensembl
## AH60773       EnsDb AHEnsDbs/v91/EnsDb.H.. http://www.ensembl.org     ensembl
## ...             ...                    ...                    ...         ...
## AH78783       EnsDb AHEnsDbs/v99/EnsDb.H.. http://www.ensembl.org     ensembl
## AH79689       EnsDb AHEnsDbs/v100/EnsDb... http://www.ensembl.org     ensembl
## AH83216       EnsDb AHEnsDbs/v101/EnsDb... http://www.ensembl.org     ensembl
## AH89180       EnsDb AHEnsDbs/v102/EnsDb... http://www.ensembl.org     ensembl
## AH89426       EnsDb AHEnsDbs/v103/EnsDb... http://www.ensembl.org     ensembl
```

```
# Acquire the latest annotation files
id <- ahDb %>%
  mcols() %>%
  rownames() %>%
  tail(n = 1)
# Download the appropriate Ensembldb database
edb <- ah[[id]]
# Extract gene-level information from database
annotations <- genes(edb, 
                     return.type = "data.frame")

#View(annotations)
# Select annotations of interest
annotations <- annotations %>%
  dplyr::select( gene_name, gene_biotype,seq_name)
# Explore biotypes
annotations$gene_biotype %>%
  factor() %>%
  levels()
```

```
##  [1] "IG_C_gene"                          "IG_C_pseudogene"                   
##  [3] "IG_D_gene"                          "IG_J_gene"                         
##  [5] "IG_J_pseudogene"                    "IG_pseudogene"                     
##  [7] "IG_V_gene"                          "IG_V_pseudogene"                   
##  [9] "lncRNA"                             "LRG_gene"                          
## [11] "miRNA"                              "misc_RNA"                          
## [13] "Mt_rRNA"                            "Mt_tRNA"                           
## [15] "polymorphic_pseudogene"             "processed_pseudogene"              
## [17] "protein_coding"                     "pseudogene"                        
## [19] "ribozyme"                           "rRNA"                              
## [21] "rRNA_pseudogene"                    "scaRNA"                            
## [23] "scRNA"                              "snoRNA"                            
## [25] "snRNA"                              "sRNA"                              
## [27] "TEC"                                "TR_C_gene"                         
## [29] "TR_D_gene"                          "TR_J_gene"                         
## [31] "TR_J_pseudogene"                    "TR_V_gene"                         
## [33] "TR_V_pseudogene"                    "transcribed_processed_pseudogene"  
## [35] "transcribed_unitary_pseudogene"     "transcribed_unprocessed_pseudogene"
## [37] "translated_processed_pseudogene"    "translated_unprocessed_pseudogene" 
## [39] "unitary_pseudogene"                 "unprocessed_pseudogene"            
## [41] "vault_RNA"
```

```
# Extract IDs for mitochondrial genes
mt <- annotations %>% 
  dplyr::filter(seq_name == "MT") %>%
  dplyr::pull(gene_name)
mt<-gsub("MT-","",mt)
# Number of UMIs assigned to mitochondrial genes
metadata$mtUMI <- Matrix::colSums(rawcounts[which(rownames(rawcounts) %in% mt),], na.rm = T)
# Ensure all NAs receive zero counts
metadata$mtUMI[is.na(metadata$mtUMI)] <- 0
# Calculate of mitoRatio per cell
metadata$mitoRatio <- metadata$mtUMI/metadata$nUMI

# Filtering steps
# Keep cells with nUMI greater than 100
idx <- which(metadata$nUMI > 100)

# Extract the counts for those cells
counts_c <- rawcounts[, idx]

# Extract the metadata for those cells
metadata_c <- metadata[idx,]

# Save data to single cell experiment variable
library(SingleCellExperiment)
se <- SingleCellExperiment(assays=list(counts=counts_c), 
                           colData = metadata_c)
# QC plots
# Create a data frame containing the metrics for visualizations
metrics <- colData(se) %>%
  as.data.frame

#save(annotations,metadata_c,counts_c,metrics,se,file="data/000_QC-metrics.rda")

# Visualize the number of cell counts per cell
require(gridExtra)
#png("plots/S1_QCs.png",w=9000,h=3000,res=600)
gp1<-metrics %>% 
  ggplot(aes(x=sample, fill=sample)) + 
  geom_bar() + 
  scale_fill_manual(values = alpha(c("gray30","sienna1", "skyblue2"), .9))+
  theme_bw()+
  ggtitle("Number of Total Cells")+
  theme(plot.title = element_text(hjust = 0.5))

# Visualize the number UMIs/transcripts per cell
gp2<-metrics %>% 
  ggplot(aes(color=sample, x=nUMI, fill= sample)) + 
 # geom_density() + 
  stat_density(geom = "line",size=3,position = "identity",alpha=0.8)+
  scale_x_log10() + 
  scale_color_manual(values=c("gray30","sienna1", "skyblue2")) +
  theme_bw()+
  ylab("log10 cell density")+
  ggtitle("Number UMI/Cell")+
  theme(plot.title = element_text(hjust = 0.5))


# Visualize the distribution of genes detected per cell via boxplot
gp3<-metrics %>% 
  ggplot(aes(x=sample, y=nGene, fill=sample)) + 
  geom_boxplot() +
  theme_bw()+
  scale_fill_manual(values = alpha(c("gray30","sienna1", "skyblue2"), .9))+
  ggtitle("Number Genes/Cell")+
  theme(plot.title = element_text(hjust = 0.5))
plot(gp1+gp2+gp3)
```

Figure S2. QC plots

```
#dev.off()
```

## 3.4 QC Metrics and low quality cells filtering

Create and print QC metrics table. Low quality cells are filtered out, data are stored in a SingleCellExperiment object.

```
splitA_tab<-metrics[metrics$sample=="SplitA",]
QC_A<-splitA_tab[,c(2:3,6,7)]
QC_A<-apply(QC_A,2,mean)
QC_A<-signif(QC_A, digits=2)

splitB_tab<-metrics[metrics$sample=="SplitB",]
QC_B<-splitB_tab[,c(2:3,6,7)]
QC_B<-apply(QC_B,2,mean)
QC_B<-signif(QC_B, digits=2)

splitM_tab<-metrics[metrics$sample=="multiplet",]
QC_M<-splitM_tab[,c(2:3,6,7)]
QC_M<-apply(QC_M,2,mean)
QC_M<-signif(QC_M, digits=2)

QC_A<-t(QC_A)
rownames(QC_A)<-"SplitA"
QC_B<-t(QC_B)
rownames(QC_B)<-"SplitB"
QC_M<-t(QC_M)
rownames(QC_M)<-"multiplet"

tab<-rbind(QC_A,QC_B,QC_M)

#png("plots/000_stat_tab.png",w=3500,h=1500,res=600)
grid.table(tab)
```

Figure S3. QC metrics table

```
#dev.off()

# Filtering low quality cells
# Filter out low quality reads using selected thresholds - these will change with experiment
# nUMI > 500
# nGene > 300
# log10GenesPerUMI > 0.75
# mitoRatio < 0.15

keep <- metrics %>%
  dplyr::filter(nUMI > 500 , 
                nGene > 300,
                log10GenesPerUMI > 0.75,
                mitoRatio < 0.15,
  ) %>% 
  pull(cells)
# Subset the cells to only include those that meet the thresholds specified
se_c <- se[ ,keep]

# Save subset to new metrics variable
metrics_clean <- colData(se_c) %>%
  as.data.frame()

filt_rawcounts<-rawcounts[,keep]
# Save cleaned single-cell experiment as .RData to load at any time
#save(se_c, filt_rawcounts, keep, file = "data/000_se_filtered.rda")
```

# 4 FPKM and TPM Normalization, Create Seurat Object

Normalization of raw count data will be performed with the FPKM and TPM algorithms and with the LogNormalize function of Seurat. Store data in a Seurat Object.

```
## Normalization
# Extract gene lengths (more precisely, transcript lengths)
fname<-"markdown/data/genelengths.rda"
if(!file.exists(fname)){
  library(GenomicFeatures)
  supportedUCSCtables(genome="hg38", url="http://genome.ucsc.edu/cgi-bin/")
  hg<-makeTxDbFromUCSC(genome="hg38",tablename="refGene")
  exonic<-exonsBy(hg,by="gene")
  redexonic<-reduce(exonic)
  genelengths<-sum(width(redexonic))
  names(genelengths)<-eg2sym(names(genelengths))
  genelengths<-genelengths[!duplicated(names(genelengths))]
  genelengths<-genelengths[genelengths>0]
  genelengths<-genelengths[!is.na(genelengths)]
  #save(genelengths,file=fname)
}else{load(fname)}

# Function to calculate FPKM (https://www.rna-seqblog.com/rpkm-fpkm-and-tpm-clearly-explained/)
fpkm<-function(counts,genelengths){
  common<-intersect(rownames(counts),names(genelengths))
  counts<-counts[common,]
  lengths<-genelengths[common]
  fpms<-apply(counts,2,function(x){1E6*x/sum(x)})
  fpkms<-fpms
  for(i in 1:nrow(fpms)){
    fpkms[i,]<-1E3*fpms[i,]/lengths[i]
  }
  return(fpkms)
}
fname<-"markdown/data/000_fpkms.rda"
if(!file.exists(fname)){
  fpkms<-fpkm(filt_rawcounts,genelengths)
  #save(fpkms,file=fname)
}
# Function to calculate TPM (https://www.rna-seqblog.com/rpkm-fpkm-and-tpm-clearly-explained/)
tpm<-function(counts,genelengths){
  common<-intersect(rownames(counts),names(genelengths))
  counts<-counts[common,]
  lengths<-genelengths[common]
  intermediate<-counts
  for(i in 1:nrow(counts)){
    intermediate[i,]<-1E3*counts[i,]/lengths[i]
  }
  tpms<-apply(intermediate,2,function(x){1E6*x/sum(x)})
  return(tpms)
}
fname<-"markdown/data/000_tpms.rda"
if(!file.exists(fname)){
  tpms<-tpm(filt_rawcounts,genelengths)
  #save(tpms,file=fname)
}

hashtags<-hashtags[keep]

## Log-Normalize with Seurat
# By default, Seurat employs a global-scaling normalization method LogNormalize that
# normalizes the gene expression measurements for each cell by the total expression,
# multiplies this by a scale factor (10,000 by default), and log-transforms the result:
# Perform Seurat normalization
seuset<-CreateSeuratObject(counts=filt_rawcounts,project="SY5Y",min.cells=3,min.features=1000)
seuset # 13927 genes, 2143 samples
```

```
## An object of class Seurat 
## 13927 features across 2143 samples within 1 assay 
## Active assay: RNA (13927 features, 0 variable features)
```

```
seuset<-NormalizeData(seuset,normalization.method="LogNormalize",scale.factor=10000)

# # Get metadata in it
samplenames<-rownames(seuset@meta.data)
commonsamples<-intersect(samplenames,names(hashtags))
tags<-hashtags[commonsamples]
seuset@meta.data$orig.ident<-as.factor(tags)
seuset <- FindVariableFeatures(seuset, selection.method = "vst", nfeatures = 2000)
# Identify the 10 most highly variable genes
top10 <- head(VariableFeatures(seuset), 10)
# plot variable features with and without labels
plot1 <- VariableFeaturePlot(seuset)
plot2 <- LabelPoints(plot = plot1, points = top10, repel = TRUE)
#png("plots/S2_variablegenes.png",w=4000,h=4000,res=600)
plot2
```

Figure S4. Standardized variance vs. Average Expression Plot

```
#dev.off()
save(seuset,hashtags,tags,file="markdown/data/000_seurat_obj.rda")
```

# 5 Explorative Analysis

We will investigate gene expression by identifying most varying and most stable genes across the dataset.

```
# ########## Descriptive plots
splitA<-as.matrix(seuset@assays$RNA@data)[,seuset@meta.data$orig.ident=="SplitA"]
splitB<-as.matrix(seuset@assays$RNA@data)[,seuset@meta.data$orig.ident=="SplitB"]
multiplets<-as.matrix(seuset@assays$RNA@data)[,seuset@meta.data$orig.ident=="multiplet"]
col_a<-"sienna1"
col_b<-"skyblue2"
# # Identify Possible new candidate housekeeping genes
# # a housekeeping should be a gene with minimum variance across cells
x<-apply(splitA,1,mean)
xvar<-apply(splitA,1,var)
# 

# # Expression of housekeeping genes by chromosome band
# ### Chromosome bands ----
fname<-"markdown/data/mlist.rda"
if(!file.exists(fname)){
  mdf<-msigdbr(species="Homo sapiens") # Retrieve all human gene sets
  mlist<-mdf %>% split(x=.$gene_symbol,f=.$gs_name)
  #save(mlist,file=fname)
}else{load(fname)}
chrom_bands <- mlist[grep("chr",names(mlist))]
mtgenes<-mlist[grep("MT",names(mlist))]
mtgenes<-mtgenes["MT"]
mtgenes[["MT"]]<-gsub("MT-","",mtgenes[["MT"]])
mito<-mtgenes[["MT"]]
mito<-c("ND1","ND2","ND3","ND4","CYTB","COX1","COX2","COX3","ATP6")
mito<-unique(mito)
mtgenes[["MT"]]<-mito
chrom_bands<-append(chrom_bands,mtgenes)
chrs<-c("chr1","chr2","chr3","chr4","chr5","chr6","chr7","chr8","chr9","chr10","chr11","chr12","chr13",
        "chr14","chr15","chr16","chr17","chr18","chr19","chr20","chr21","chr22","chrX","chrY","MT")
```

## 5.1 Descriptive Plots

```
# Variance vs. Expression plots
#png("plots/Fig_SplitA.png",w=4000,h=4000,res=600)
scatter(x,xvar,showLine = FALSE,
        grid = FALSE,subtitle = "",pch=20,xlim=c(0,6),col=col_a,xlab="LogNorm Average Expression",ylab="Average Variance",
        main="SY5Y Split A")
genes<-names(x[x>4])
genes2<-names(xvar[xvar<0.2])
gene<-intersect(genes,genes2)
housekeeping<-c("ACTB","GAPDH","B2M")
housekeeping<-c(housekeeping,gene)
housekeeping<-unique(housekeeping)
textplot3(x[housekeeping],xvar[housekeeping],words = housekeeping,cex=0.8,font=2,line.col = "grey")
```

Figure S5. Average Variance vs. LogNorm Average Expression in SplitA

```
#dev.off()
```

```
# whole dataset
total<-cbind(splitA,splitB)
t<-apply(total,1,mean)
tvar<-apply(total,1,var)
#png("plots/Fig_SplitAll.png",w=4000,h=4000,res=600)
scatter(t,tvar,showLine = FALSE,
        grid = FALSE,subtitle = "",pch=20,xlim=c(0,6),col="lightpink",xlab="LogNorm Average Expression",ylab="Average Variance",
        main="SY5Y Split A + Split B")
genes<-names(t[t>4])
genes2<-names(tvar[tvar<0.2])
gene<-intersect(genes,genes2)
housekeeping<-c("ACTB","GAPDH","B2M")
housekeeping<-c(housekeeping,gene)
thousekeeping<-unique(housekeeping)
textplot3(t[thousekeeping],tvar[thousekeeping],words = housekeeping,cex=0.8,font=2,line.col = "grey")
```

Figure S6. Average Variance vs. LogNorm Average Expression in SplitA + SplitB

```
#dev.off()
```

```
y<-apply(splitB,1,mean)
yvar<-apply(splitB,1,var)
#png("plots/Fig_SplitB.png",w=4000,h=4000,res=600)
scatter(y,yvar,showLine = FALSE,
        grid = FALSE,subtitle = "",pch=20,xlim=c(0,6),col=col_b,xlab="LogNorm Average Expression",ylab="Average Variance",
        main="SY5Y Split B")
genes<-names(y[y>4])
genes2<-names(yvar[yvar<0.2])
gene<-intersect(genes,genes2)
housekeeping<-c("ACTB","GAPDH","B2M")
housekeeping<-c(housekeeping,gene)
housekeeping<-unique(housekeeping)
textplot3(x[housekeeping],xvar[housekeeping],words = housekeeping,cex=0.8,font=2,line.col = "grey")
```

Figure S7. Average Variance vs. LogNorm Average Expression in SplitB

```
#dev.off()
```

## 5.2 HK genes plot in SY5Y

Plot selected genes characterized by high expression and low variance. These genes are good housekeeping candidates.

```
# Find housekeeping genes coordinates
housekeeping<-sort(thousekeeping)
HK<-c()
for (gene in housekeeping){
  for(i in 1:length(chrom_bands)){
    band<-names(chrom_bands)[i]
    genes_here<-chrom_bands[[band]]
    if(gene%in%genes_here){
      message(gene," is in ",band)
      tpm<-c(gene,band)
      HK<-rbind(HK,tpm)
    }
  }
}
rownames(HK)<-HK[,1]
colnames(HK)<-c("Gene","Band")

# ACTB is in chr7p22
# B2M is in chr15q21
# COX1 is in MT
# COX2 is in MT
# COX3 is in MT
# CYTB is in MT
# EEF1A1 is in chr6q13
# GAPDH is in chr12p13
# ND4 is in MT
# RPL10 is in chrXq28
# RPL13A is in chr19q13
# RPS18 is in chr6p21
# RPS2 is in chr16p13

# Coordinates
cols<-"royalblue4"
means<-c()
for (i in 1:nrow(HK)){
  sub<-chrom_bands[grep(HK[i,2],names(chrom_bands))]
  genes_here<-sub[[HK[i,2]]]
  genes_here<-intersect(HK[i,1],genes_here)
  allexp<-t[genes_here]
  mean<-mean(allexp)
  means<-c(means,mean)
  names(means)[length(means)]<-HK[i,2]
}
#means<-log10(means)
#png("plots/000_HK_bars.png",w=3000,h=3000,res=600)
bp<-barplot(means,col=cols,ylab="LogNorm Average Exp",xaxt="n",main="HK gene expression in SY5Y",ylim=c(0,6))
for(i in 1:nrow(HK)){
  chr<-HK[i,2]
  xwhere<-mean(bp[i])
  text(xwhere,5.5,labels=HK[i,1],font=2,cex=0.7,srt=45,offset = -0.3)
}
axis(1,at=bp,labels=names(means),cex.axis=1,las=2)
```

Figure S8. Average Expression of HK genes in SY5Y

```
#dev.off()

t[housekeeping]
```

```
##     ACTB      B2M     COX1     COX2     COX3     CYTB   EEF1A1    GAPDH 
## 2.296830 1.067794 4.803187 5.192140 5.082765 4.390471 4.056871 3.132416 
##      ND4    RPL10   RPL13A    RPS18     RPS2 
## 4.636691 4.200807 4.383413 4.673808 4.574188
```

```
tvar[housekeeping]
```

```
##      ACTB       B2M      COX1      COX2      COX3      CYTB    EEF1A1     GAPDH 
## 0.4903847 0.6059069 0.1759359 0.1768072 0.1838019 0.1792094 0.1191639 0.2176918 
##       ND4     RPL10    RPL13A     RPS18      RPS2 
## 0.1563155 0.1895634 0.1876353 0.1543088 0.1992206
```

```
ratio<-t/tvar

# ratio graph
png("markdown/plots/000_HK_bars_ratio.png",w=3000,h=3000,res=600)
bp<-barplot(ratio[housekeeping],col=cols,ylab="LogNorm Average Exp / Variance",xaxt="n",main="HK Exp/Var ratio in SY5Y cells",ylim=c(0,50))
for(i in 1:nrow(HK)){
  chr<-HK[i,2]
  xwhere<-mean(bp[i])
  text(xwhere,40,labels=HK[i,1],font=2,cex=0.7,srt=45,offset = -0.3)
}
axis(1,at=bp,labels=names(means),cex.axis=1,las=2)
dev.off()
```

```
## png 
##   2
```

```
# other candidates
range(ratio)
```

```
## [1]  0.3131295 37.8213617
```

```
names(ratio[ratio>15])
```

```
##  [1] "RPL11"  "H3-3A"  "RPS7"   "RPL37A" "RPL15"  "RPL9"   "RPS3A"  "RPS14" 
##  [9] "RACK1"  "RPS18"  "RPL10A" "EEF1A1" "RPS4X"  "RPL10"  "RPL30"  "RPL8"  
## [17] "RPS6"   "RPL35"  "RPL7A"  "RPLP2"  "RPL27A" "FAU"    "RPS3"   "RPS25" 
## [25] "RPL41"  "RPL6"   "RPLP0"  "RPLP1"  "RPS2"   "RPL13"  "RPL19"  "RPS21" 
## [33] "RPS15"  "RPS16"  "RPS19"  "RPL18"  "RPL13A" "RPS9"   "RPL28"  "RPS5"  
## [41] "RPL3"   "ND1"    "ND2"    "COX1"   "COX2"   "ATP6"   "COX3"   "ND4"   
## [49] "CYTB"
```

```
rat<-ratio[ratio>15]
housekeeping<-names(rat)

# Find housekeeping genes coordinates
housekeeping<-sort(housekeeping)
HK<-c()
for (gene in housekeeping){
  for(i in 1:length(chrom_bands)){
    band<-names(chrom_bands)[i]
    genes_here<-chrom_bands[[band]]
    if(gene%in%genes_here){
      message(gene," is in ",band)
      tpm<-c(gene,band)
      HK<-rbind(HK,tpm)
    }
  }
}
rownames(HK)<-HK[,1]
colnames(HK)<-c("Gene","Band")

# ATP6 is in MT
# COX1 is in MT
# COX2 is in MT
# COX3 is in MT
# CYTB is in MT
# EEF1A1 is in chr6q13
# FAU is in chr11q13
# H3-3A is in chr1q42
# ND1 is in MT
# ND2 is in MT
# ND4 is in MT
# RACK1 is in chr5q35
# RPL10 is in chrXq28
# RPL10A is in chr6p21
# RPL11 is in chr1p36
# RPL13 is in chr16q24
# RPL13A is in chr19q13
# RPL15 is in chr3p24
# RPL18 is in chr19q13
# RPL19 is in chr17q12
# RPL27A is in chr11p15
# RPL28 is in chr19q13
# RPL3 is in chr22q13
# RPL30 is in chr8q22
# RPL35 is in chr9q33
# RPL37A is in chr2q35
# RPL41 is in chr12q13
# RPL6 is in chr12q24
# RPL7A is in chr9q34
# RPL8 is in chr8q24
# RPL9 is in chr4p14
# RPLP0 is in chr12q24
# RPLP1 is in chr15q23
# RPLP2 is in chr11p15
# RPS14 is in chr5q33
# RPS15 is in chr19p13
# RPS16 is in chr19q13
# RPS18 is in chr6p21
# RPS19 is in chr19q13
# RPS2 is in chr16p13
# RPS21 is in chr20q13
# RPS25 is in chr11q23
# RPS27A is in chr2p16
# RPS3 is in chr11q13
# RPS3A is in chr4q31
# RPS4X is in chrXq13
# RPS5 is in chr19q13
# RPS6 is in chr9p22
# RPS7 is in chr2p25
# RPS9 is in chr19q13

# ratio graph
png("markdown/plots/000_HK_bars_ratio_complete.png",w=7000,h=3000,res=600)
bp<-barplot(ratio[housekeeping],col=cols,ylab="LogNorm Average Exp / Variance",xaxt="n",main="HK Exp/Var ratio in SY5Y cells",ylim=c(0,50))
for(i in 1:nrow(HK)){
  chr<-HK[i,2]
  xwhere<-mean(bp[i])
  text(xwhere,45,labels=HK[i,1],font=2,cex=0.7,srt=45,offset = -0.3)
}
axis(1,at=bp,labels=HK[,2],cex.axis=1,las=2)
dev.off()
```

```
## png 
##   2
```

## 5.3 Dimensionality reduction and cell cycle score regression

```
# Variance vs. Expression plots
#png("plots/003_var_vs_exp.png",w=3000,h=3000,res=300)
load("markdown/data/000_seurat_obj.rda")

# Data Scaling (seurat method default)
all.genes <- rownames(seuset)
seuset <- ScaleData(seuset, features = all.genes)
seuset <- RunPCA(seuset, features = VariableFeatures(object = seuset))
#png("mdpi/plots/001_PCA.png",w=4000,h=4000,res=600) 
gp1<-DimPlot(object=seuset, reduction="pca",group.by = "orig.ident",cols = c("gray30","sienna1", "skyblue2"))+
  ggtitle("")
 
#dev.off()

# Meaningful PCs
## Determine the optimal number of significant PCs

## Determine the optimal number of significant PCs for subsequent clustering
## Determine percent of variation associated with each PC
pct<-seuset@reductions$pca@stdev / sum(seuset@reductions$pca@stdev) * 100
## Calculate cumulative percents for each PC
cum<-cumsum(pct)
## Determine which PC exhibits cumulative percent greater than 90% and % variation associated with the PC as less than 5
co1<-which(cum>90&pct<5)[1]
## Determine the difference between variation of PC and subsequent PC
co2 <- sort(which((pct[1:length(pct)-1] - pct[2:length(pct)]) > 0.1),  decreasing = T)[1] + 1 # last point where change of % of variation is more than 0.1%.
co2 # 12 PCs
```

```
## [1] 12
```

```
## Minimum of the two calculation
pcs <- min(co1, co2) # change to any other number
pcs # 12
```

```
## [1] 12
```

```
# Regress out cell cycle and plot it
# A list of cell cycle markers, from Tirosh et al, 2015, is loaded with Seurat.  We can
# segregate this list into markers of G2/M phase and markers of S phase
s.genes <- cc.genes$s.genes
g2m.genes <- cc.genes$g2m.genes
regressed <- CellCycleScoring(seuset, s.features = s.genes, g2m.features = g2m.genes, set.ident = TRUE)
regressed <- RunPCA(regressed, features = c(s.genes, g2m.genes))
gp2<-DimPlot(object=regressed)+
  ggtitle("")
# Remove CC by signals that separate non-cycling cells and cycling cells maintained, 
# but differences in cell cycle phase among proliferating cells will be regressed out of the data
regressed$CC.Difference <- regressed$S.Score - regressed$G2M.Score
regressed <- ScaleData(regressed, vars.to.regress = "CC.Difference", features = rownames(regressed))
regressed <- RunPCA(regressed, features = c(s.genes, g2m.genes))
#png("mdpi/plots/001_PCA.png",w=4000,h=4000,res=600) 
gp3<-DimPlot(object=regressed, reduction="pca")+
  ggtitle("")
#png("mdpi/plots/Fig_2.png",w=6500,h=2500,res=600) 
plot_grid(gp1+gp2+gp3)+
  ggtitle("PCA")+
  theme(plot.title = element_text(hjust = 0.5))
```

Figure S9. Principal Component Analysis (left), before (centre) and after cell cycle score partial regression (right)

```
#dev.off()
#save(seuset,regressed,file="data/001_seurat_objects.rda")

# Differential Expression in Seurat
levels(seuset)
```

```
## [1] "SY5Y"
```

```
seuset <- SetIdent(seuset, value = seuset@meta.data$orig.ident)
levels(seuset)
```

```
## [1] "multiplet" "SplitA"    "SplitB"
```

```
split.de.markers <- FindMarkers(seuset, ident.1 = "SplitA", ident.2 = "SplitB")
# view results
head(split.de.markers)
```

```
##           p_val avg_log2FC pct.1 pct.2 p_val_adj
## MT2A 0.01130562 -0.3963513 0.271 0.322         1
## VGF  0.07046914 -0.2586887 0.611 0.634         1
## MT1X 0.42317777 -0.3492205 0.261 0.243         1
```

```
# ######    p_val avg_log2FC pct.1 pct.2 p_val_adj
# MT2A 0.01130562 -0.3963513 0.271 0.322         1
# VGF  0.07046914 -0.2586887 0.611 0.634         1
# MT1X 0.42317777 -0.3492205 0.261 0.243         1
levels(regressed)
```

```
## [1] "G2M" "G1"  "S"
```

```
regressed <- SetIdent(regressed, value = regressed@meta.data$orig.ident)
levels(regressed)
```

```
## [1] "multiplet" "SplitA"    "SplitB"
```

```
split.de.markers <- FindMarkers(regressed, ident.1 = "SplitA", ident.2 = "SplitB")
# view results
head(split.de.markers)
```

```
##           p_val avg_log2FC pct.1 pct.2 p_val_adj
## MT2A 0.01130562 -0.3963513 0.271 0.322         1
## VGF  0.07046914 -0.2586887 0.611 0.634         1
## MT1X 0.42317777 -0.3492205 0.261 0.243         1
```

```
#            p_val avg_log2FC pct.1 pct.2 p_val_adj
# MT2A 0.01130562 -0.3963513 0.271 0.322         1
# VGF  0.07046914 -0.2586887 0.611 0.634         1
# MT1X 0.42317777 -0.3492205 0.261 0.243         1
```

# 6 Monocle Clustering and Analysis

```
###### Monocle3 transfer of seurat object
library(monocle3)
library(SeuratWrappers)
# subset cells excluding multiplets
seusub<-subset(x = regressed, subset = orig.ident != "multiplet")
cds <- as.cell_data_set(seusub)
## Calculate size factors using built-in function in monocle3
cds <- estimate_size_factors(cds)
## Add gene names into CDS
cds@rowRanges@elementMetadata@listData[["gene_short_name"]] <- rownames(seusub[["RNA"]])
## Step 1: Normalize and pre-process the data
cds <- preprocess_cds(cds, num_dim = 50)
cds <- reduce_dimension(cds)
#plot_cells(cds)
#png("mdpi/plots/0001_monocle3_graph1.png",w=3000,h=3000,res=400)
gp1<-plot_cells(cds,color_cells_by="orig.ident",label_cell_groups = FALSE,cell_size = 1.5)+
  scale_color_manual(values=c("sienna1", "skyblue2")) 
cds <- cluster_cells(cds, resolution=1e-3)
gp2<-plot_cells(cds,cell_size = 1.5,group_label_size = 5)
cds<-learn_graph(cds)
#plot_cells(cds)

gp3<-monocle3::plot_cells(cds,
                     color_cells_by = "cluster",
                     label_groups_by_cluster=FALSE,
                     label_leaves=FALSE,
                     label_branch_points=FALSE,
                     cell_size = 1.5, label_cell_groups = FALSE)
marker_test_res <- top_markers(cds, group_cells_by="cluster", 
                               reference_cells=1000, cores=1)
#save(marker_test_res,file = "results/001_cluster_DE.rda")
#print(marker_test_res %>% group_by(cell_group) %>% top_n(5, avg_logFC) %>% data.frame)

top_specific_markers <- marker_test_res %>%
  dplyr::filter(fraction_expressing >= 0.95) %>%
  dplyr::filter(marker_score >= 0.25) %>%
  group_by(cell_group) %>%
  top_n(3, pseudo_R2)


top_specific_marker_ids <- unique(top_specific_markers %>% pull(gene_id))

gp4<-plot_genes_by_group(cds,
                         top_specific_marker_ids,
                         group_cells_by="cluster",
                         ordering_type="maximal_on_diag",
                         max.size=3)

#png("mdpi/plots/001_monocle3_graph2.png",w=5500,h=4500,res=600)
plot_grid(gp1+gp2+gp3+gp4)
```

Figure S10. Monocle3 Analysis

```
#dev.off()

#Finding genes that change as a function of pseudotime (should it be performe d before?)
test_res <- monocle3::graph_test(cds, neighbor_graph="principal_graph", cores=1)
# #Finding genes that are involved in the transition between the start point and the end point
monocle_qvalues <- test_res$q_value
names(monocle_qvalues) <- rownames(test_res)
monocle_best_genes <- names(sort(monocle_qvalues))[1:1000]
#save(cds,monocle_best_genes,monocle_qvalues,file="data/001_cds_monocle3.rda")
#dev.off()
```

```
# Dissection of heterogeneity
# We used the f-scLVM method (implemented by the Bioconductor slalom package) 
# and the pathway annaotation from MSigDb v7.2/WikiPathways
# to scout for annotated and not-annotated sources of heterogeneity within the dataset.
# 
# ##### WARNING: since this will take several hours, code is given as commented
# library(slalom)
# load("data/000_seurat_obj.rda")
# # Data Scaling (seurat method default)
# all.genes <- rownames(seuset)
# seuset <- ScaleData(seuset, features = all.genes)
# seusub<-subset(x = seuset, subset = orig.ident != "multiplet")
# dim(seusub@assays$RNA@data)
# expmat<-as.matrix(seusub@assays$RNA@data)
# sce<-SingleCellExperiment::SingleCellExperiment(assays=list(logcounts=expmat))
# # We will supply f-scLVM with genesets in a GeneSetCollection object.
# # We will use a curated annotation as suggested in the f-scLCM vignette
# # The following file was downloaded from MSigDB on Jan 20, 2021
# gmtfile<-"data/c2.cp.wikipathways.v7.2.symbols.gmt"
# genesets<-GSEABase::getGmt(gmtfile)
# # Generate a f-scLVM model
# model<-newSlalomModel(sce,genesets,min_genes = 30)
# # 211 annotated factors retained;  376 annotated factors dropped.
# # 4266  genes retained for analysis.
# # Initialize it
# model<-initSlalom(model)
# # Train it
# model<-trainSlalom(model,nIterations=10000,minIterations = 3000) # model converged after 3050 iterations
# png("plots/002_slalom_Relevance.png",w=6000,h=3000,res=600)
# plotRelevance(model)
# dev.off()
# png("plots/002_slalom_Terms.png",w=4000,h=3000,res=600)
# plotTerms(model)
# dev.off()
# png("plots/002_slalom_Copper.png",w=3000,h=3000,res=600)
# plotLoadings(model,"WP_COPPER_HOMEOSTASIS")
# dev.off()
# png("plots/002_slalom_TFs.png",w=3000,h=3000,res=600)
# plotLoadings(model,"WP_TRANSLATION_FACTORS")
# dev.off()
# png("plots/002_slalom_GLYCO.png",w=3000,h=3000,res=600)
# plotLoadings(model,"WP_GLYCOLYSIS_AND_GLUCONEOGENESIS")
# dev.off()
# png("plots/002_slalom_05.png",w=3000,h=3000,res=600)
# plotLoadings(model,"hidden05")
# dev.off()
# png("plots/002_slalom_04.png",w=3000,h=3000,res=600)
# plotLoadings(model,"hidden04")
# dev.off()
# png("plots/002_slalom_03.png",w=3000,h=3000,res=600)
# plotLoadings(model,"hidden03")
# dev.off()
# png("plots/002_slalom_02.png",w=3000,h=3000,res=600)
# plotLoadings(model,"hidden02")
# dev.off()
# terms<-topTerms(model)
# str(terms)
# write.xlsx2(terms,file="mdpi/results/002_slalom_topterms.xlsx")
# png("plots/002_slalom_cycle.png",w=3000,h=3000,res=600)
# plotLoadings(model,"WP_CELL_CYCLE")
# dev.off()
#  # add result to single_cell experiment
# sce <- addResultsToSingleCellExperiment(sce, model) # store results in a SingleCell Experiment Object
# save(sce,file="results/002_slalom_sce.rda")
```

# 7 Building a co-expression network

We will use the SY5Y single-cell transcriptomes (n = 2143) to build a TF-Target network with the corto algorithm.

```
load("markdown/data/000_seurat_obj.rda")
load("tfs_2020.rda")
library(corto)
expmat<-as.matrix(seuset@assays$RNA@data)
#dim(expmat) # 13927  2143
regulon<-corto(expmat,centroids = centroids,nbootstraps = 1000,nthreads = 6,p = 1E-20)
save(regulon,file="markdown/results/regulon.rda")

# visualize the regulon in Cytoscape
fname<-"markdown/results/network.txt"
cat("Regulator\tTarget\tMoA\tlikelihood\n",file=fname)
for(tf in names(regulon)){
  sub<-regulon[[tf]]
  toprint<-cbind(
    rep(tf,length(sub$tfmode)),
    names(sub$tfmode),
    sub$tfmode,
    sub$likelihood
  )
  write.table(toprint,file=fname,append=TRUE,col=FALSE,row=FALSE,quote=FALSE,sep="\t")
}
# load the network.txt file in Cytoscape for visualization
```

# 8 Technical Session Info

The following code describes the R environment used to generate this document and will help making it fully reproducible should there be future updates in any of the packages.

```
sessionInfo()
```

```
## R version 4.0.4 (2021-02-15)
## Platform: x86_64-w64-mingw32/x64 (64-bit)
## Running under: Windows 10 x64 (build 19042)
## 
## Matrix products: default
## 
## locale:
## [1] LC_COLLATE=Italian_Italy.1252  LC_CTYPE=Italian_Italy.1252   
## [3] LC_MONETARY=Italian_Italy.1252 LC_NUMERIC=C                  
## [5] LC_TIME=Italian_Italy.1252    
## system code page: 65001
## 
## attached base packages:
## [1] stats4    parallel  stats     graphics  grDevices utils     datasets 
## [8] methods   base     
## 
## other attached packages:
##  [1] ensembldb_2.14.0            AnnotationFilter_1.14.0    
##  [3] wordcloud_2.6               RColorBrewer_1.1-2         
##  [5] org.Rn.eg.db_3.12.0         org.Mm.eg.db_3.12.0        
##  [7] biomaRt_2.46.3              org.Hs.eg.db_3.12.0        
##  [9] SeuratWrappers_0.3.0        monocle3_0.2.3.0           
## [11] ggforce_0.3.3               slalom_1.12.0              
## [13] SingleCellExperiment_1.12.0 SummarizedExperiment_1.20.0
## [15] MatrixGenerics_1.2.1        matrixStats_0.58.0         
## [17] devtools_2.3.2              usethis_2.0.1              
## [19] GenomicFeatures_1.42.1      AnnotationDbi_1.52.0       
## [21] Biobase_2.50.0              GenomicRanges_1.42.0       
## [23] GenomeInfoDb_1.26.2         IRanges_2.24.1             
## [25] S4Vectors_0.28.1            AnnotationHub_2.22.0       
## [27] BiocFileCache_1.14.0        dbplyr_2.1.0               
## [29] BiocGenerics_0.36.0         gridExtra_2.3              
## [31] msigdbr_7.2.1               corto_1.1.4                
## [33] dplyr_1.0.5                 cowplot_1.1.1              
## [35] ggplot2_3.3.3               SeuratObject_4.0.0         
## [37] Seurat_4.0.0               
## 
## loaded via a namespace (and not attached):
##   [1] rappdirs_0.3.3                rtracklayer_1.50.0           
##   [3] scattermore_0.7               coda_0.19-4                  
##   [5] tidyr_1.1.3                   bit64_4.0.5                  
##   [7] knitr_1.31                    irlba_2.3.3                  
##   [9] DelayedArray_0.16.2           data.table_1.14.0            
##  [11] rpart_4.1-15                  RCurl_1.98-1.2               
##  [13] generics_0.1.0                RhpcBLASctl_0.20-137         
##  [15] callr_3.6.0                   leidenbase_0.1.2             
##  [17] RSQLite_2.2.3                 RANN_2.6.1                   
##  [19] proxy_0.4-25                  future_1.21.0                
##  [21] bit_4.0.4                     spatstat.data_2.0-0          
##  [23] xml2_1.3.2                    lubridate_1.7.10             
##  [25] httpuv_1.5.5                  assertthat_0.2.1             
##  [27] viridis_0.5.1                 xfun_0.21                    
##  [29] hms_1.0.0                     jquerylib_0.1.3              
##  [31] evaluate_0.14                 promises_1.2.0.1             
##  [33] fansi_0.4.2                   progress_1.2.2               
##  [35] caTools_1.18.1                igraph_1.2.6                 
##  [37] DBI_1.1.1                     htmlwidgets_1.5.3            
##  [39] RcppArmadillo_0.10.2.1.0      spdep_1.1-5                  
##  [41] purrr_0.3.4                   ellipsis_0.3.1               
##  [43] RSpectra_0.16-0               annotate_1.68.0              
##  [45] deldir_0.2-10                 sparseMatrixStats_1.2.1      
##  [47] vctrs_0.3.6                   remotes_2.2.0                
##  [49] ROCR_1.0-11                   abind_1.4-5                  
##  [51] RMariaDB_1.1.0                cachem_1.0.4                 
##  [53] withr_2.4.1                   grr_0.9.5                    
##  [55] sctransform_0.3.2             GenomicAlignments_1.26.0     
##  [57] prettyunits_1.1.1             goftest_1.2-2                
##  [59] cluster_2.1.1                 lazyeval_0.2.2               
##  [61] crayon_1.4.1                  units_0.7-0                  
##  [63] slam_0.1-48                   pkgconfig_2.0.3              
##  [65] labeling_0.4.2                tweenr_1.0.1                 
##  [67] nlme_3.1-152                  pkgload_1.2.0                
##  [69] ProtGenerics_1.22.0           rlang_0.4.10                 
##  [71] globals_0.14.0                lifecycle_1.0.0              
##  [73] miniUI_0.1.1.1                rsvd_1.0.3                   
##  [75] rprojroot_2.0.2               polyclip_1.10-0              
##  [77] lmtest_0.9-38                 graph_1.68.0                 
##  [79] Matrix_1.3-2                  raster_3.4-5                 
##  [81] boot_1.3-27                   zoo_1.8-9                    
##  [83] Matrix.utils_0.9.8            ggridges_0.5.3               
##  [85] processx_3.5.1                png_0.1-7                    
##  [87] viridisLite_0.3.0             bitops_1.0-6                 
##  [89] KernSmooth_2.23-18            Biostrings_2.58.0            
##  [91] speedglm_0.3-3                blob_1.2.1                   
##  [93] DelayedMatrixStats_1.12.3     classInt_0.4-3               
##  [95] stringr_1.4.0                 parallelly_1.23.0            
##  [97] scales_1.1.1                  memoise_2.0.0                
##  [99] GSEABase_1.52.1               magrittr_2.0.1               
## [101] plyr_1.8.6                    ica_1.0-2                    
## [103] gplots_3.1.1                  gdata_2.18.0                 
## [105] zlibbioc_1.36.0               compiler_4.0.4               
## [107] plotrix_3.8-1                 fitdistrplus_1.1-3           
## [109] Rsamtools_2.6.0               cli_2.3.1                    
## [111] LearnBayes_2.15.1             XVector_0.30.0               
## [113] listenv_0.8.0                 patchwork_1.1.1              
## [115] pbapply_1.4-3                 ps_1.6.0                     
## [117] MASS_7.3-53.1                 mgcv_1.8-34                  
## [119] tidyselect_1.1.0              stringi_1.5.3                
## [121] highr_0.8                     yaml_2.2.1                   
## [123] askpass_1.1                   ggrepel_0.9.1                
## [125] pbmcapply_1.5.0               grid_4.0.4                   
## [127] sass_0.3.1                    tools_4.0.4                  
## [129] future.apply_1.7.0            BH_1.75.0-0                  
## [131] farver_2.1.0                  Rtsne_0.15                   
## [133] digest_0.6.27                 BiocManager_1.30.10          
## [135] shiny_1.6.0                   Rcpp_1.0.6                   
## [137] BiocVersion_3.12.0            later_1.1.0.1                
## [139] RcppAnnoy_0.0.18              httr_1.4.2                   
## [141] sf_0.9-7                      colorspace_2.0-0             
## [143] XML_3.99-0.5                  fs_1.5.0                     
## [145] tensor_1.5                    reticulate_1.18              
## [147] splines_4.0.4                 uwot_0.1.10                  
## [149] expm_0.999-6                  spatstat.utils_2.0-0         
## [151] sp_1.4-5                      spData_0.3.8                 
## [153] plotly_4.9.3                  sessioninfo_1.1.1            
## [155] xtable_1.8-4                  jsonlite_1.7.2               
## [157] spatstat_1.64-1               testthat_3.0.2               
## [159] R6_2.5.0                      gmodels_2.18.1               
## [161] pillar_1.5.1                  htmltools_0.5.1.1            
## [163] mime_0.10                     glue_1.4.2                   
## [165] fastmap_1.1.0                 BiocParallel_1.24.1          
## [167] class_7.3-18                  interactiveDisplayBase_1.28.0
## [169] codetools_0.2-18              pkgbuild_1.2.0               
## [171] utf8_1.1.4                    lattice_0.20-41              
## [173] bslib_0.2.4                   tibble_3.1.0                 
## [175] curl_4.3                      leiden_0.3.7                 
## [177] gtools_3.8.2                  openssl_1.4.3                
## [179] survival_3.2-7                limma_3.46.0                 
## [181] rmarkdown_2.7                 desc_1.3.0                   
## [183] munsell_0.5.0                 e1071_1.7-4                  
## [185] GenomeInfoDbData_1.2.4        reshape2_1.4.4               
## [187] gtable_0.3.0
```
